# The physiological functions of the Cbp2D and Cbp2E proteins are important for insoluble cellulose-dependent growth in *Cellvibrio japonicus*

**DOI:** 10.1128/aem.00818-25

**Published:** 2025-09-04

**Authors:** Baily E. Kakacek, Jiabao Liang, Kyle A. Dickerson, Jeffrey G. Gardner

**Affiliations:** 1 Department of Biological Sciences, University of Maryland - Baltimore County 12265, Baltimore, Maryland, USA; University of Nebraska-Lincoln, Lincoln, Nebraska, USA

**Keywords:** CAZyme, cellodextrin, cellulase, cellulose, *Cellvibrio japonicus*, polysaccharide degradation

## Abstract

Microbial deconstruction of plant polysaccharides is important for environmental nutrient cycling, and bacteria proficient at this process have extensive suites of polysaccharide-specific enzymes. In the gram-negative saprophyte *Cellvibrio japonicus*, genome annotation suggests that 17 genes are predicted to encode Carbohydrate-Active enZymes (CAZymes) with roles in cellulose degradation; however, previous work suggested that only a subset of these genes is essential. Building upon that work, here, we identify the required and minimally sufficient set of enzymes for complete degradation of cellulose using a combination of transcriptomics, gene deletion analysis, heterologous expression studies, and metabolite analysis. We identified six CAZyme-encoding genes required for cellulose deconstruction in *C. japonicus*, which are *cel3B*, *cel5B*, *cel6A*, *lpmo10B*, *cbp2D*, and *cbp2E*. These genes encode for a β-glucosidase, an endoglucanase, a cellobiohydrolase, a lytic polysaccharide mono-oxygenase, and two carbohydrate-binding proteins, respectively. These CAZyme-encoding genes are essential for growth using insoluble cellulose by *C. japonicus* and sufficient for using soluble cellulose when heterologously expressed in *Escherichia coli*. Moreover, during *C. japonicus* growth using insoluble cellulose, we detected no cellodextrins in the medium, which suggested that cello-oligosaccharide uptake is highly efficient. RNA-seq analysis corroborates these results as we observed several genes significantly upregulated during growth using cellulose that encode TonB-dependent and ABC transporters. Our revised model of cellulose utilization by *C. japonicus* suggests a greater importance for the Cbp2D and Cbp2E proteins than previously thought and that rapid cellodextrin uptake by *C. japonicus* is a mechanism to maximize the energetic return on investment for the production and secretion of CAZymes.

**IMPORTANCE:**

Bacterial contributions to fixed carbon turnover in the soil are increasingly being shown as vital to global nutrient cycling. Additionally, the discovery and characterization of bacterial polysaccharide deconstruction enzymes are fundamental for industrial and biomedical applications, such as the production of renewable fuels, sustainable detergents, and nutritional supplements. Our analysis of *C. japonicus* cellulose deconstruction is significant because it provides a roadmap for analyzing a suite of enzymes that may be functionally redundant and identifies those that are truly essential. Specifically, our results indicate that the functions of the Cbp2D and Cbp2E proteins, previously thought to have only a supporting or accessory role, in fact play a more direct part in cellulose deconstruction in *C. japonicus*. Furthermore, our results suggest that a strategy where recovery of soluble oligosaccharides is a priority to maximize the energetic economics for polysaccharide degradation in *C. japonicus*.

## INTRODUCTION

In the environment, microbial action is responsible for the decomposition of plant material (1), and this recycling of carbon and energy is carried out by synergistic communities of bacteria and fungi (2). The power of microbial polysaccharide deconstruction is also being harnessed for biotechnology and biomedical advancement. For these dual reasons of ecological understanding and industrial innovation, considerable research has gone into the identification and characterization of bacteria and fungi proficient at polysaccharide deconstruction, along with their enzyme complements. The most abundant polysaccharide is cellulose (3), which is a linear polymer of glucose units attached by β-1,4 linkages (4). Individual cellulose chains are capable of hydrogen bonding to one other, contributing to the structural rigidity needed for plant cell walls and increasing crystallinity as more chains hydrogen bond to one another (5). The length of chains contributes to different properties in plant structures. For example, shorter cellulose chains contribute to denser materials such as wood or bark, and longer chains are responsible for more flexible materials such as cotton (6).

The biological method of cellulose breakdown is mediated by the action of Carbohydrate-Active enZymes (CAZymes) (7). The largest class of CAZymes are those belonging to the Glycosidic Hydrolase (GH) category, which are further classified according to substrate specificity, relative positions of action in the polysaccharide chain, and the stereochemical outcome of the reaction they are involved in (8). Currently, there are 189 unique GH families, with those having cellulase-specific members found in families GH 5, 6, 7, 8, 9, 12, 26, 44, 45, 48, 61, and 74 (9). Despite this diversity, a key characteristic of many GHs is their modular structure; a catalytic domain specific to a particular polysaccharide is often linked with one or more carbohydrate-binding modules (CBMs). There are three major types of CBMs (Types A, B, and C) that are further sub-classified into over 60 families, where categorization is based on the size, shape, and sugar composition of substrates bound (10, 11). When part of a GH, one or more CBMs can either act as either structural support or serve to modify the catalytic domain’s activity (12). Current nomenclature conventions for proteins that have CBMs but undefined enzymatic functions report them as carbohydrate-binding proteins (Cbps) and categorize them using the CBM (e.g., Cbp2D has a CBM2).

Cellulolytic bacteria are diverse; however, the presence of more than three cellulose-specific CAZyme-encoding genes suggests a species may be able to utilize recalcitrant cellulose (13). Only relatively few species are able to utilize crystalline cellulose, and it has been proposed that a species should only be considered “truly cellulolytic” if it is able to utilize crystalline cellulose. Bacteria that are proficient at polysaccharide deconstruction often have dozens to hundreds of CAZyme-encoding genes, which suggest a specialization for polysaccharide utilization in the environment. One such bacterium with over 200 CAZyme-encoding genes is the gram-negative soil saprophyte *Cellvibrio japonicus* (14), originally named *Pseudomonas fluorescens* sub. *cellulosa* (15) and renamed due to its genomic similarity to *Cellvibrio mixtus* (16). Most of the CAZyme-encoding genes in *C. japonicus* are predicted to belong to GH families, and specific to this study, there are 17 genes predicted to be cellulose-specific (14). In a 2013 review, the cellulase complement of *C. japonicus* was compared to 16 other cellulose-degrading bacteria (17). It was argued that *C. japonicus* has an unsurprising set of GH5 and GH9 cellulases but was notable for not possessing any GH48 proteins, which are considered important for cellulose deconstruction in many gram-positive bacteria. It was hypothesized in this review that the mechanism of cellulose depolymerization was different in *C. japonicus* compared to common gram-positive models from the *Clostridium* or *Caldicellulosiruptor* genera. From a 2021 review that summarized the carbon and nitrogen acquisition strategies of four polysaccharide-degrading bacteria (*Bacteroides thetaiotaomicron*, *Cellvibrio japonicus*, *Clostridium thermocellum*, and *Caldicellulosiruptor bescii*), the total number of predicted CAZymes found in *C. japonicus* (100 GH-encoding genes from 47 different families) is less than that of *B. thetaiotaomicron* (300 GH-encoding genes from 62 different families), but more than *C. thermocellum* (79 GH-encoding genes from 29 different families) or *C. bescii* (63 GH-encoding genes from 30 different families) (18). This trend held true for the number of GH families uniquely represented in the four bacteria, with *C. japonicus* being the only one to have enzymes from the GH4, 6, 19, 37, 45, 46, 62, 98, 103, and 128 families. Notable for this report are the GH6 and GH45, which are the GH families known to have cellulase activities.

The current model of cellulose degradation in *C. japonicus* (19) contains secreted endo- and exoglucanases, encoded in particular by *cel5B* and *cel6A,* respectively, as being released into the external space. Also released into the external space is Lpmo10B, a lytic polysaccharide mono-oxygenase from the AA10 CAZyme family (20). While this model is the culmination of substantial genomic, transcriptomic, genetic, and biochemical research, there are still gaps in our understanding of cellulose utilization by *C. japonicus*. Specifically, the enzymes involved in the initial stages of cellulose degradation, the generation of cello-oligosaccharides, and the mechanism by which expected short-chain cellodextrins are imported into the periplasmic and cytoplasmic spaces are not yet fully understood. The current model emphasizes the importance of the Cel5B, Cel6A, and Lpmo10B enzymes for polymer deconstruction and the Cel3B enzyme for cellobiose cleavage (21, 22). However, this model is incomplete as it does not address to what extent there is functional redundancy among the predicted eight GH5 and three GH9 enzymes in *C. japonicus* or identify the electron source for the LPMO. Furthermore, there is no consideration for how soluble cellodextrins are transported into the bacterium.

To address these knowledge gaps, in this study, we determined the necessity and sufficiency of the entire suite of cellulose-specific CAZyme-encoding genes for *C. japonicus*. We used a combination of transcriptomics and gene deletion analysis to identify the six genes that are essential for cellulose deconstruction. These analyses emphasized the importance of the Cbp2D and Cbp2E proteins and uncovered a surprising number of TonB-dependent transporters. While a previous study in *C. japonicus* identified the Cbp2D and Cbp2E proteins as potentially having electron-transfer activities for Lpmo10B, a definitive function could not be identified using a combination of *in vivo* and *in vitro* methods (21). In addition to the CBM2 domain, the Cbp2D protein is predicted to have and X158 domain with homology to a YceI-like ubiquinone-8 domain and an X183 *c*-type cytochrome domain. A structural study of the X183 domain from Cbp2D found that it had redox potential; however, the physiological function of the full protein remained unclear (23). Consequently, the importance of this current study highlights a thus-far overlooked CAZyme complement found in many polysaccharide-degrading bacteria, specifically the Cbps, and how they may have direct roles in cellulose (or other polysaccharide) cleavage. The discovery of a high number of nutrient transporters led us to investigate cellodextrin utilization by *C. japonicus*, where we uncovered a highly efficient uptake system. This study reinforces the idea that broadly classified carbohydrate-binding proteins can be major contributors to cellulose deconstruction and are a likely reservoir of novel activities. Moreover, our results further the hypothesis that nutrient recovery is a powerful driver of bacterial evolution as a mechanism to balance the energetic economics for recalcitrant polysaccharide utilization.

## MATERIALS AND METHODS

### Bacterial strains, media, and growth conditions

Wild-type *Cellvibrio japonicus* strain Ueda107 was acquired from the National Collections of Industrial, Marine, and Food Bacteria [NCIMB #10462]. *Escherichia coli* strain W was obtained from the American Type Culture Collection (ATCC #9637). The complete list of strains and plasmids used in this study is compiled in Table S1. For propagation, both *E. coli* and *C. japonicus* strains were grown using 3-(N-morpholino) propanesulfonic acid (MOPS) minimal media (24), supplemented with 0.2% (wt:vol) glucose (catalog number: G0520, Teknova). For growth experiments using cellulosic substrates, MOPS medium was supplemented with cellobiose (0.5%, wt:vol) (catalog number: AC108460250, Acros), carboxymethyl-cellulose (1%, wt:vol) (catalog number: 150560, MP Biomedicals), Avicel (1%, wt:vol) (catalog number: 11365-1KG, Millipore Sigma), or filter paper (1%, wt:vol) (catalog number: 1703965, BioRad). Production of phosphoric acid swollen cellulose (PASC) and hydrochloric acid swollen cellulose (HASC) used methods published previously without modification (25, 26). Plate media were made with the addition of 1.5% (wt:vol) agar. All growth media were autoclave-sterilized using a steam cycle (121°C at 16 psi for 30 min). Growth experiments using insoluble cellulose were performed with sterile biomass containment devices and agar capture systems as needed, as described previously (27). When required, kanamycin (catalog number: 25389-94-0, VWR) was added at a concentration of 50 µg/mL. Cells were grown at 30°C with high aeration (225 RPM) as done previously (28). Cell growth was measured with a Spectronic 20D + or an EPOCH2 microplate reader at OD_600_ (28), and all experiments were performed in biological triplicate. Growth analyses were visualized using the GraphPad Prism software package with default settings.

### RNA-seq analysis

Transcriptomic experiments, including biological triplicate growth measurements and cell samplings, were conducted as published previously (21, 22). During the mid-exponential phase, a sample of 45 mL was collected and added to 5 mL of ice cold 5% (vol:vol) acidic phenol:absolute ethanol to stop cellular metabolism. Cells were then centrifuged (8,000 × *g* for 5 minutes at 4°C), after which the supernatant was removed and the pellet snap-frozen in a dry ice/ethanol bath for 5 minutes. The samples were then stored at −80°C. Frozen cell pellets were sent to GeneWIZ (South Plainfield, NJ) for extraction, processing, and RNA sequencing on a fee-for-service basis. An Illumina HiSeq2000 was used with a 1 × 50 bp configuration with ~10M reads per sample. The sequencing data, which included FASTQ and QC files, were returned for analysis by our group. The web-based system Galaxy (29) was used to analyze the FASTQ single-end read files generated from sequencing. FASTQ files were checked for quality using the FastQC tool (30), with any reads of a Phred score of >30 (less than one error per 1,000 base calls) being usable. The quality-assured FASTQ reads were then mapped onto the *C. japonicus* reference genome retrieved from ENSEMBLE using the HISAT2 tool (31). Reads for this study were unstranded and therefore did not need to be specified according to the tool’s parameters. Alignment summaries with SAM/BAM files reading at over 80% coverage were used for next steps. The aligned SAM/BAM files for each condition were then analyzed using the HTSeq-count tool (32) with the *C. japonicus* reference genome in the .gtf file format. Outputs obtained from HTSeq-count were subsequently used for analysis in DESeq2 (33) to determine differential gene expression. Published RNA-seq data for *C. japonicus* grown using glucose (accession: GSE90955) was used for comparative expression analysis, as done previously (22). The tabular file output of the DESeq2 tool was aligned to an annotated gene database for *C. japonicus* based on the initial characterization (14) to obtain names and functions for all genes. Volcano and MA plots were generated using GraphPad Prism software as done previously (34, 35).

### Genetic methods

Protocols were followed according to previous studies for the construction of *C. japonicus* in-frame gene deletion mutants (36). Briefly, 500 base pair areas upstream and downstream from the gene targeted for deletion were amplified using PCR. A culture of *E. coli* pK18*mobsacB* (37) was grown using lysogeny broth (38, 39) and the plasmid extracted using a QIAprep Spin Miniprep Kit (catalog no: 27106) according to the manufacturer’s instructions. The purified vector was cut with EcoRI-HF and HindIII-HF restriction enzymes (catalog numbers: R3101S and R3104S, New England Biolabs) and recovered using a QIAquick PCR Purification Kit (catalog no: 28104). The PCR fragments were then assembled onto the pK18*mobsacB* plasmid using NEBuilder HiFi DNA Assembly Master Mix (catalog no: E2621L) and then transformed into chemically competent *E. coli* cells by heat shock and screened for the correctly assembled inserts as done previously (40). Correctly assembled plasmids were recovered and transferred into *E. coli* S17 and used in tri-parental mating, as previously described (20, 35, 41). PCR-verified *C. japonicus* mutant strains were frozen as permanent stocks at −80°C. A complete list of all genes studied in this report that includes name, locus ID, CAZyme family classification, enzymatic activity known (or predicted), and UniProt accession number is found in Table S4.

For heterologous expression studies in *E. coli*, plasmids containing *C. japonicus* CAZyme-encoding genes were designed using SnapGene version 4.1.5 software, and sequences of *C. japonicus* were retrieved from NCBI. Four separate plasmids were designed using variants of the pUC18 plasmid (42): pCEL1 contained *C. japonicus cel3B*; pCEL3 contained *C. japonicus cel6A*, *cel3B*, and *cel5B*; pCEL6 contained *C. japonicus cbp2E*, *cbp2D*, *lpmo10B*, *cel6A*, *cel3B*, and *cel5B*; and pCEL- was an empty vector to serve as the negative control. Plasmids were synthesized by GeneWIZ and then transformed into competent *E. coli* W cells using the heat-shock method.

### Congo Red staining

The method for Congo Red staining followed established protocols (43). Briefly, overnight cultures of wild-type and Δ*gsp C. japonicus* and *E. coli* W/pCEL1, /pCEL3, /pCEL6, and /pCEL- strains were used to spot 10 μL on a 1% (wt:vol) CMC MOPS agar plate. All spots were dried prior to incubation at 30°C for either 48 or 96 hours. After incubation, each plate was stained with 5 mL 0.1% aqueous Congo red dye (catalog number: 2250-2, Ricca) for 10 minutes at room temperature. After decanting excess dye, 5 mL of a 1M sodium chloride (catalog number: S640-500, Fisher Scientific) counter-stain was added to each plate for an additional 10 minutes. Plates were allowed to dry overnight at room temperature and were visualized with a light box.

### Cell-free extract generation and thin-layer chromatography

Biological triplicate cultures of wild-type *C. japonicus* were grown using 1% wt/vol filter paper with biomass containment devices as described above. When cultures reached the early exponential phase (OD_600_: 0.2), and then 50 µL of 50 mg/mL kanamycin was added to each tube and allowed to incubate for 48 hours, with continued growth measurements being taken to ensure no further increase in OD_600_. Next, two 1 mL samples were removed from each culture tube and filter-sterilized using a 0.2 µm filter (catalog number: 50-104-9912, Fisher Scientific) and the supernatants stored at 4°C and used within 1 week. Acid hydrolysis was performed on one of the filtered supernatants using 24N H_2_SO_4,_ while the other remained untreated. Sugars in the supernatants were resolved using 20 cm × 10 cm silica gel aluminum plates (catalog number: 1055540001). The mobile phase consisted of a ratio of 5:3:1 2-propanol (catalog number: A416-1, Fisher Scientific): ethanol: water, as described previously (40). After TLC chamber equilibration (30 minutes), the solid phase was spotted with 3 µL of 10 mM standards of glucose (1.8 mg/mL) (catalog number: D16-500, Fisher Scientific), cellobiose (3.4 mg/mL) (catalog number: AC108460250, Acros), cellotriose (5.1 mg/mL) (catalog number: O-CTR-50MG, Megazyme), cellotetraose (6.66 mg/mL) (catalog number: O-CTE-50MG, Megazyme), cellopentaose (8.3 mg/mL) (catalog number: O-CPE-20MG, Megazyme), and cellohexaose (9.9 mg/mL) (catalog number: O-CHE, Megazyme). A 200 µL sample (applied in 10 µL aliquots to prevent spreading) of filtered supernatants was then spotted alongside the cellodextrin standards and allowed to dry completely. The plate was placed in the development chamber for approximately 4 hours or until the solvent front was 2 cm from the top of the plate. The plate was removed from the development chamber and allowed to dry completely in a chemical fume hood for 5–10 minutes. A visualization solution of 5% (vol:vol) 18 N sulfuric acid (catalog number: SX1244Y-1, Millipore Sigma) to 95% methanol (catalog number: LC168004, LabChem) was poured into a shallow glass tray in a chemical fume hood. Once the silica plate had dried fully, it was dipped in the visualization solution and then removed and allowed to dry in a chemical fume hood. Finally, the plate was placed in an 120°C oven for 5–7 minutes to visualize the cellodextrins. When quantitation of glucose-equivalents from a cellodextrin sample was required, acid hydrolysis was performed using 24N H_2_SO_4_ to reduce all cellodextrins to glucose, and a Glucose Assay Kit (catalog number: GAGO20-1KT; Sigma-Aldrich) was used according to the manufacturer’s instructions.

### Cellodextrin consumption rate assay

Biological triplicate cultures of wild-type *C. japonicus* were grown in a 96-well plate using MOP medium supplemented with 11 mM glucose (0.2% wt:vol), 5.5 mM cellobiose (0.19% wt:vol), or 1.8 mM cellohexaose (0.18% wt:vol) for 15 or 24 hours, as described above. Then, a 60 µL sample was removed from each cellobiose and cellohexaose culture well and incubated with 3 µL β-glucosidase from *Agrobacterium* sp. (Megazyme; catalog number: E-BGOSAG) at 50°C for 1 hour. After enzyme incubation to reduce cellodextrins to glucose, a Glucose Assay Kit was used according to the manufacturer’s instructions to quantitate the remaining glucose in the samples.

### Glucose uptake assay

Biological triplicate cultures of wild-type *C. japonicus* and *E. coli* W were grown in a 96-well plate for 24 hours using MOP medium supplemented with decreasing glucose concentrations: 2,000 µg/mL, 1,000 µg/mL, 500 µg/mL, 250 µg/mL, 125 µg/mL, 63 µg/mL, 31 µg/mL, and 0 µg/mL. Then, a 40 µL sample was removed from each culture, and a Glucose Assay Kit was used according to the manufacturer’s instructions. For quantitation of the initial glucose concentration, MOP media with glucose concentration ranging from 125 µg/mL to 2,000 µg/mL were diluted by 5×, 10×, 20×, or 50× with distilled water.

## RESULTS

### Transcriptomics analysis identified significantly upregulated genes that encode CAZymes and oligosaccharide transporters in response to growth using cellulose

Previous gene expression studies in *C. japonicus* used either promoter fusions or DNA microarrays to identify genes responsive to changes in the substrate (21, 44). Relevant to the current study, the microarray study found that *C. japonicus* broadly upregulated CAZyme-encoding genes in response to Avicel; however, there were also some genes that also were upregulated in response to the growth rate. What was surprising was that despite there being 17 predicted cellulase-encoding genes in *C. japonicus*, there did not appear to be a core set of shared genes for pair-wise comparisons between glucose grown and Avicel grown cells during the exponential growth and stationary phase. In the report, *C. japonicus* growth on Avicel appeared to be biphasic, which was explained as due to the microcrystalline nature of Avicel, where the amorphous regions were degraded first and the crystalline regions attacked later. Given that naturally occurring cellulose is predominantly crystalline, we wanted to reevaluate *C. japonicus* gene expression for a more recalcitrant form of cellulose. Consequently, we performed RNAseq on wild-type *C. japonicus* while it was grown on filter paper.

**Fig 1:**
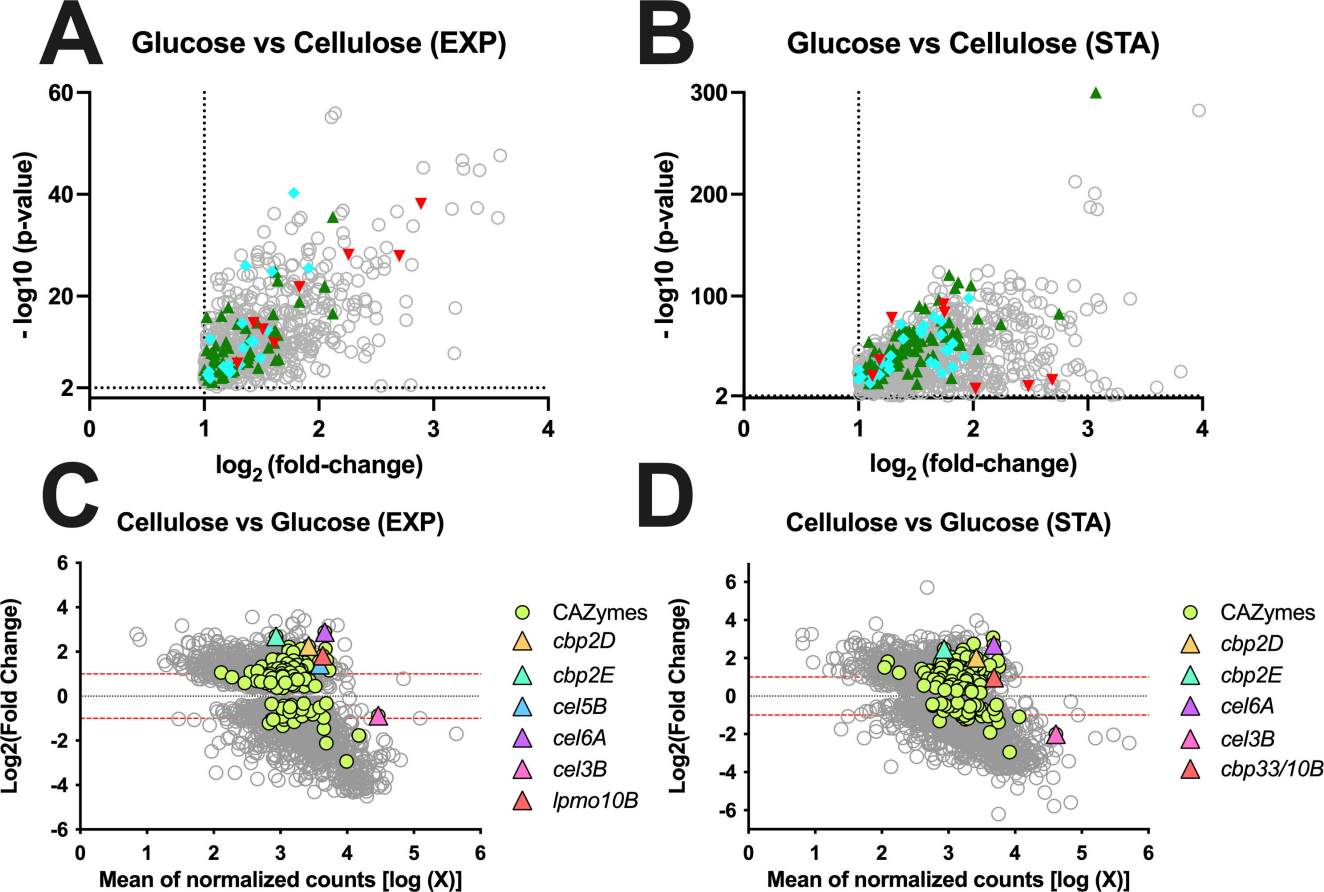
Volcano plots (panels A and B) and MA plots (panels C and D) displaying comparative expression data of wild-type *C. japonicus* grown using either glucose or filter paper as the sole carbon source. For volcano plots, fold-change (log_2_) is on the X-axis and *P*-value (−log_10_) is on the Y-axis. Dashed lines depict user-defined significance cut-offs. (**A**) Exponentially growing cells using glucose versus exponentially growing cells using insoluble cellulose. Only the 753 genes that are upregulated under cellulose conditions are shown. (**B**) Stationary-phase cells using glucose versus stationary-phase cells using insoluble cellulose. Only the 883 genes that are upregulated under cellulose conditions are shown. For panels A and B, each gray circle represents a *C. japonicus* gene. The cyan diamonds, green triangles, and red inverted triangles depict genes that encode TonB-dependent transporters (TBDTs), carbohydrate-active enzymes (CAZymes), and cellulases, respectively. MA scatterplots displaying differential expression data of wild-type *C. japonicus* grown using either glucose or filter paper as the sole carbon source. Fold-change (log_2_) is on the Y-axis, and mean of normalized counts (log) is on the X-axis. Genes with a *P*-value of ≥0.01 are excluded from the plot. Dashed lines depict log_2_ fold-change thresholds of −1 and 1. (**C**) Exponentially growing cells using glucose versus exponentially growing cells using filter paper. (**D**) Stationary-phase cells using glucose versus stationary-phase cells using filter paper. Each open circle represents a *C. japonicus* gene, and each green closed circle represents a CAZyme-encoding gene.

For the filter paper RNA-seq, we collected samples during the mid-exponential growth (exp) and at early stationary phase (sta) and used published data for glucose-grown cells (22) to perform three pair-wise comparisons that included cellulose (exp) vs glucose (exp), cellulose (sta) vs glucose (sta), and cellulose (exp) vs cellulose (sta). An analysis of the cellulose (exp) vs glucose (exp) comparison found that 753 of genes were significantly (*P*-value < 0.01) upregulated on cellulose (Fig. 1A), and among these were 64 CAZyme-encoding genes (Table S2). A wide array of CAZyme-encoding genes were upregulated despite cellulose being the sole carbon source, but importantly six cellulase-encoding genes were observed (*lpmo10B*, *cel45A*, *cel5B*, *cel5G*, *cel6A*, and *cel9A*). In addition, there were nine genes annotated as encoding carbohydrate-binding proteins (*cbp2D*, *cbp2E*, *cbp26A*, *cbp2F*, *cbp35B*, *cbp35C*, *cbp6A*, *cbp6B*, and *cbp6C*). Interestingly, nine of these genes were previously identified in DNA microarray studies of *C. japonicus* growth on Avicel (21). Also of note among the upregulated genes were 19 genes predicted to encode nutrient transporters from the TonB-dependent family. MA plot analysis of glucose-grown cells and cellulose-grown cells found that the significantly upregulated CAZyme-encoding genes on cellulose were also strongly expressed (high mean of normalized counts) for exponential growth compared to glucose. The MA plot of cellulose (exp) versus glucose (exp) identified 2,263 *C. japonicus* genes with a significant *P*-value of <0.01, and among these were 163 CAZyme-encoding genes that included *cbp2D*, *cbp2E*, *cel5B*, cel6A, and *lpmo10B* (Fig. 1C). An analysis of the cellulose (sta) vs glucose (sta) comparison found that 883 of genes were significantly (*P*-value <0.01) upregulated on cellulose (Fig. 1B). Among these were 89 CAZyme-encoding genes, with six being cellulose-specific (*cel6A*, *cel3C*, *cel45A*, *cel5E*, *cel5G*, and *cel9A*), in addition to the *cbp2D* and *cbp2E* genes (Table S3). Four of these genes were observed as upregulated under similar conditions in the previous Avicel study. There were 28 genes predicted to encode TonB-dependent transporters. An MA plot of cellulose (sta) versus glucose (sta) identified 3,151 *C. japonicus* genes with a significant *P*-value of < 0.01, and among these were 190 CAZyme-encoding that included *cel6A*, *lpmo10B*, *cbp2D*, and *cbp2E* (Fig. 1D). Finally, we performed an analysis of the cellulose (exp) vs cellulose (sta) to determine if there were any CAZyme-encoding genes under growth-rate dependent regulation. However, for this comparison, we did not observe any CAZyme-encoding genes significantly upregulated (log_2_ fold-change >2) on cellulose during the stationary phase, and only two genes upregulated overall, CJA_2120 and CJA_2729, which are predicted to encode an integral membrane protein and a pillin, respectively (Fig. S1). This contrasts with the findings of the previous Avicel study, where six cellulase-encoding genes were upregulated and likely reflects the transition from amorphous to crystalline cellulose during degradation. MA plot analysis of the cellulose (exp) versus stat (sta) found that despite no significantly upregulated CAZyme-encoding genes being observed, there were six CAZyme-encoding genes with high expression level; however, only two are denoted as encoding cellulases (*cel3B* and *cel5B*) (Fig. S1B).

### Six CAZyme-encoding genes are the minimal set required for complete cellulose deconstruction in *C. japonicus*

Previous work on *C. japonicus* cellulose degradation indicated the importance of the Cel5B and Cel6A enzymes (21); however, a comprehensive mutational study of all 17 predicted *C. japonicus* cellulase-specific CAZymes had not been completed. To determine to what extent functional redundancy plays in *C. japonicus* cellulose deconstruction, we generated in-frame gene deletions for all cellulase-encoding genes, except the β-glucosidases that have already been comprehensively studied (22, 45), and assessed growth phenotypes on insoluble cellulose (filter paper).

**Fig 2:**
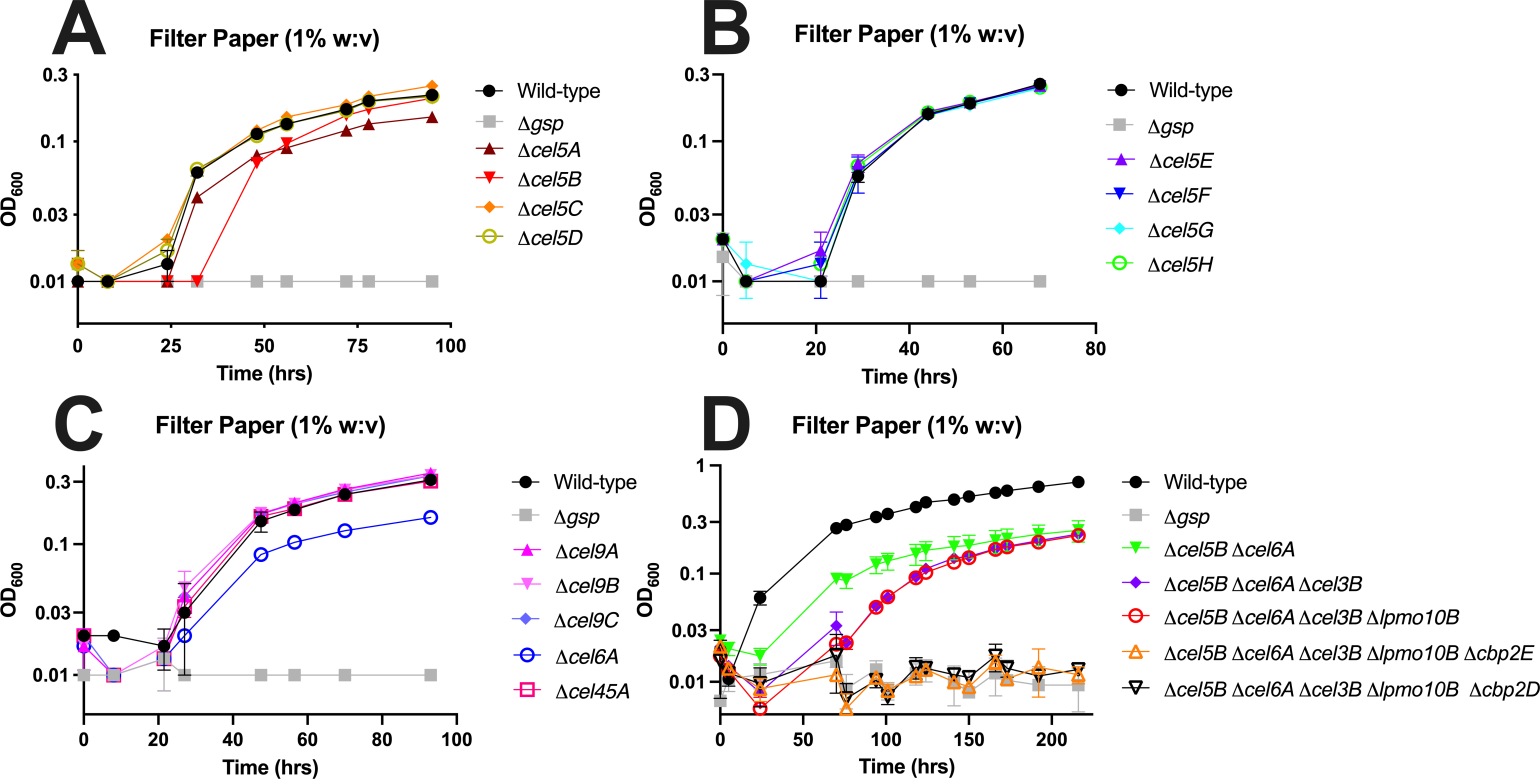
Growth analysis of *C. japonicus* mutant strains grown using insoluble cellulose as the sole carbon source. (**A**) Single mutants of the *cel5A-D* genes; (**B**) single mutants of the *cel5E-H* genes; (**C**) single mutants of the *cel9A-C*, *cel45*, and *cel6A* genes; (**D**) multi-mutant strains. In all panels, wild-type and a Δ*gsp* mutant are show as positive and negative controls, respectively. All growth experiments were performed in biological triplicate. Error bars represent standard deviation, but in many cases may be too small to be seen.

Growth data shown in Fig. S2 revealed that all strains grew as expected and like wild-type on glucose, which provided evidence that strains with multiple genes deleted did not have a general reduction in fitness. When the strains were grown using filter paper, the previously observed phenotypes for Δ*cel5A*, Δ*cel5B,* and Δ*cel6A* mutants were reproducible (Fig. 2A through C). Specifically, the Δ*cel5A* mutant had a 30% reduced final OD_600_ compared to wild-type (0.15 compared to 0.21), and the Δ*cel6A* mutant had a 50% reduced final OD_600_ compared to wild-type (0.16 compared to 0.31). The Δ*cel5B* mutant had an increased lag phase of 32 hours and a 16% reduced growth rate compared to wild-type (0.16 gen/hour compared to 0.19 gen/hour). The subtle differences between the current study and those previously published are likely due to alterations in growth conditions. Specifically, previous work did not use biomass containment, which has been shown to improve growth experiments with insoluble substrates (46, 47). As expected, a Δ*gsp* mutant, which is deficient in the general secretory pathway, is completely unable to grow using cellulose, as had been shown previously (36). Interestingly, all other cellulase-deficient single-mutant strains grew like wild-type using cellulose, suggesting that in isolation they are of lesser importance than those single mutants with growth defects. A previous study examined a limited subset of *C. japonicus* genes predicted to be important for cellulose utilization and found that while absence of a Δ*lpmo10B* strain did not have a growth defect, a Δ*cbp2D* or Δ*cbp2E* single mutant had an increased lag phase, reduced growth rate, and lower maximum growth compared to WT (21). We replicated these experiments and observed comparable results for the single mutants, specifically that the Δ*lpmo10B* mutant grew similar to WT and the D*cbp2D* or Δ*cbp2E* single mutant had reproducible growth defects, most notably a pronounced lag phase (Fig. S3).

The next series of growth experiments used strains that had combinations of cellulase-encoding genes deleted to identify those essential for cellulose deconstruction. In addition to the *cel5B* and *cel6A* genes, some strains also included *cel3B*, which encodes a β-glucosidase that has a high specificity for cellobiose. The *lpmo10B*, *cbp2D*, and *cbp2E* genes were also included based on the enzymatic activity of the former gene and the upregulation on the filter paper of the latter two genes. As shown in Fig. 2D, increasingly complex mutant strains were constructed and then grown in insoluble cellulose to identify growth defects. A Δ*cel5B* Δ*cel6A* double mutant had a protracted lag phase (~24 hours) during which there was a slight reduction in OD_600_, but a similar growth rate to wild-type between 24 and 70 hours. However, the Δ*cel5B* Δ*cel6A* double mutant ultimately had a 65% lower final OD_600_ compared to wild-type (0.25 compared to 0.71). A Δ*cel5A* Δ*cel6A* Δ*cel3B* triple mutant had an initial drop of OD_600_ over the first 50 hours of growth but then recovered to attain a maximum OD_600_ that was similar to that of the Δ*cel5B* Δ*cel6A* double mutant. Interestingly, the addition of a Δ*lpmo10B* mutation did not result in growth dynamics different from the triple mutant. The most striking results were observed when a Δ*cbp2D* or Δ*cbp2E* mutation was added to the quadruple mutant. Specifically, both the Δ*cel5A* Δ*cel6A* Δ*cel3B* Δ*lpmo10B* Δ*cbp2D* and Δ*cel5A* Δ*cel6A* Δ*cel3B* Δ*lpmo10B* Δ*cbp2E* strains were completely unable to grow using insoluble cellulose, which mirrors the phenotype observed for a Δ*gsp* mutant.

### Heterologous expression of *C. japonicus* cellulase-encoding genes enables growth using cellobiose and soluble cellulose in *E. coli*

To determine if the minimally required genes for cellulose deconstruction in *C. japonicus* could also serve as the minimally sufficient genes for the same process, they were next expressed in *E. coli* W (48). The selection of *E. coli* W was intentional due to the ability of this strain to stably harbor large plasmids of up to 100 kb (48) and its use in industrial settings (49).

A series of three plasmids were constructed from a derivative of pUC18 (50) that had a kanamycin cassette and with all genes being expressed from the P*lac* promoter. The pCEL1 vector contained only the *C. japonicus cel3B* gene; the pCEL3 vector contained the *C. japonicus cel5B*, *cel3B*, and *cel6A* genes in that order; and the pCEL6 vector contained the *C. japonicus cel6A*, *cbp2E*, *cbp2D*, *lpmo10B*, *cel3B*, and *cel5B* genes in that order (Fig. S4).

**Fig 3:**
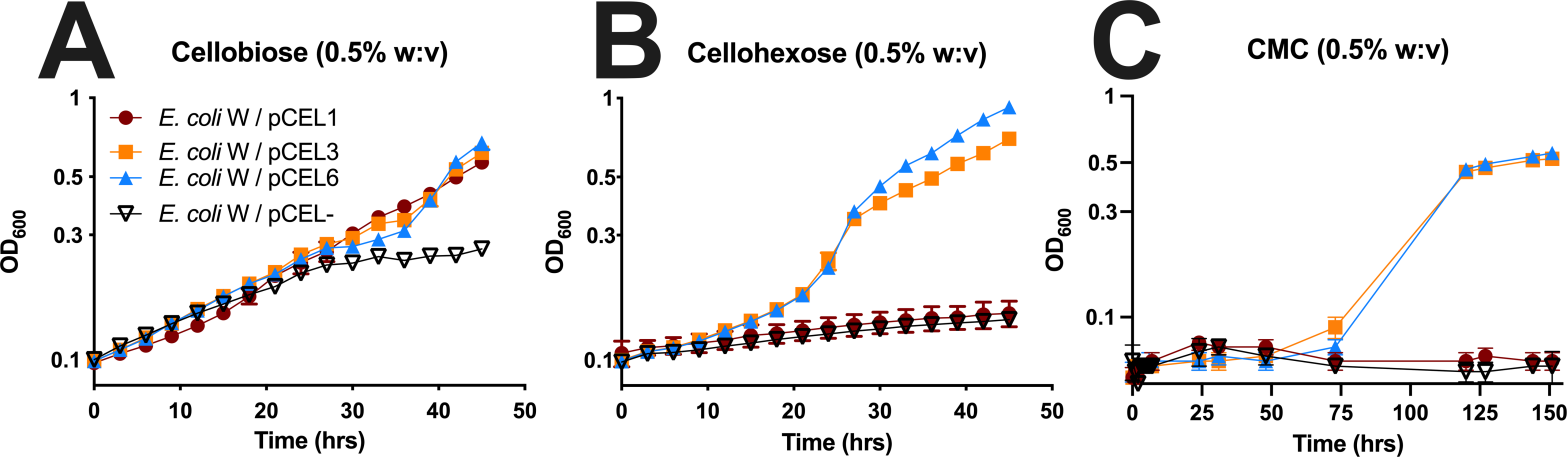
Growth analysis of engineered *E. coli* W strains expressing *C. japonicus* genes that encode cellulases during growth on cellulosic substrates. (**A**) Cellobiose; (**B**) cellohexose; (**C**) carboxymethyl cellulose (CMC). A vector-only control (VOC, pCEL-) is also shown for each condition tested. All growth experiments were performed in biological triplicate. Error bars represent standard deviation, but in many cases may be too small to be seen.

As expected, all plasmid-harboring strains were able to grow well on glucose and achieved maximum OD_600_ by 15 hours (Fig. S5). When grown using cellobiose as the sole carbon source, the strains with pCEL1, pCEL3, or pCEL6 were able to grow to an OD_600_ slightly over 1.0 (Fig. 3A). Interestingly, the growth of these strains appeared to be biphasic; however, this may be due to the fact that native *E. coli* W has some capacity to use cellobiose as a carbon source (48), which we also observed. When tested on the largest soluble cellodextrin (cellohexaose), the pCEL1 strain was unable to grow, but the pCEL3 and pCEL6 strains grew well and achieved OD_600_ slightly over 1.0 (Fig. 3B). When grown using carboxymethylcellulose (CMC), which is a soluble derivative of cellulose, the vector-only control (VOC) strain was unable to grow, which was expected (Fig. 3C). Additionally, the pCEL1 strain, which only expressed the *cel3B* β-glucosidase-encoding gene, was also unable to grow. In contrast, the pCEL3 and pCEL6 strains, which contain the *cel5B* endoglucanase-encoding gene and the *cel6A* cellbiohydrolase-encoding gene, were able to grow after a substantial lag phase (~60 hours).

The next series of growth experiments used insoluble cellulose as the sole carbon source, specifically filter paper and two types of acid-pretreated microcrystalline cellulose (Avicel), phosphoric acid swollen cellulose (PASC), and hydrochloric acid swollen cellulose (HASC). For all three substrates, none of the pCEL-harboring *E. coli* W strains were able to grow (Fig. S6). The addition of glutathione, which has previously been used to increase the activity of LPMOs, did not improve growth under any condition (data not shown). For comparison, the growth of wild-type *C. japonicus* using PASC was considerably poorer, both in terms of the growth rate and maximum OD_600_, than on filter paper. Regardless of insoluble cellulose type, we observed that *C. japonicus* had a sharp drop in OD_600_ in the first few hours before recovering, with the best growth using filter paper and HASC (FP max. OD_600_ of 0.66; HASC max. OD_600_ of 0.76), and poorer growth using PASC (max. OD_600_ of 0.35).

**Fig 4:**
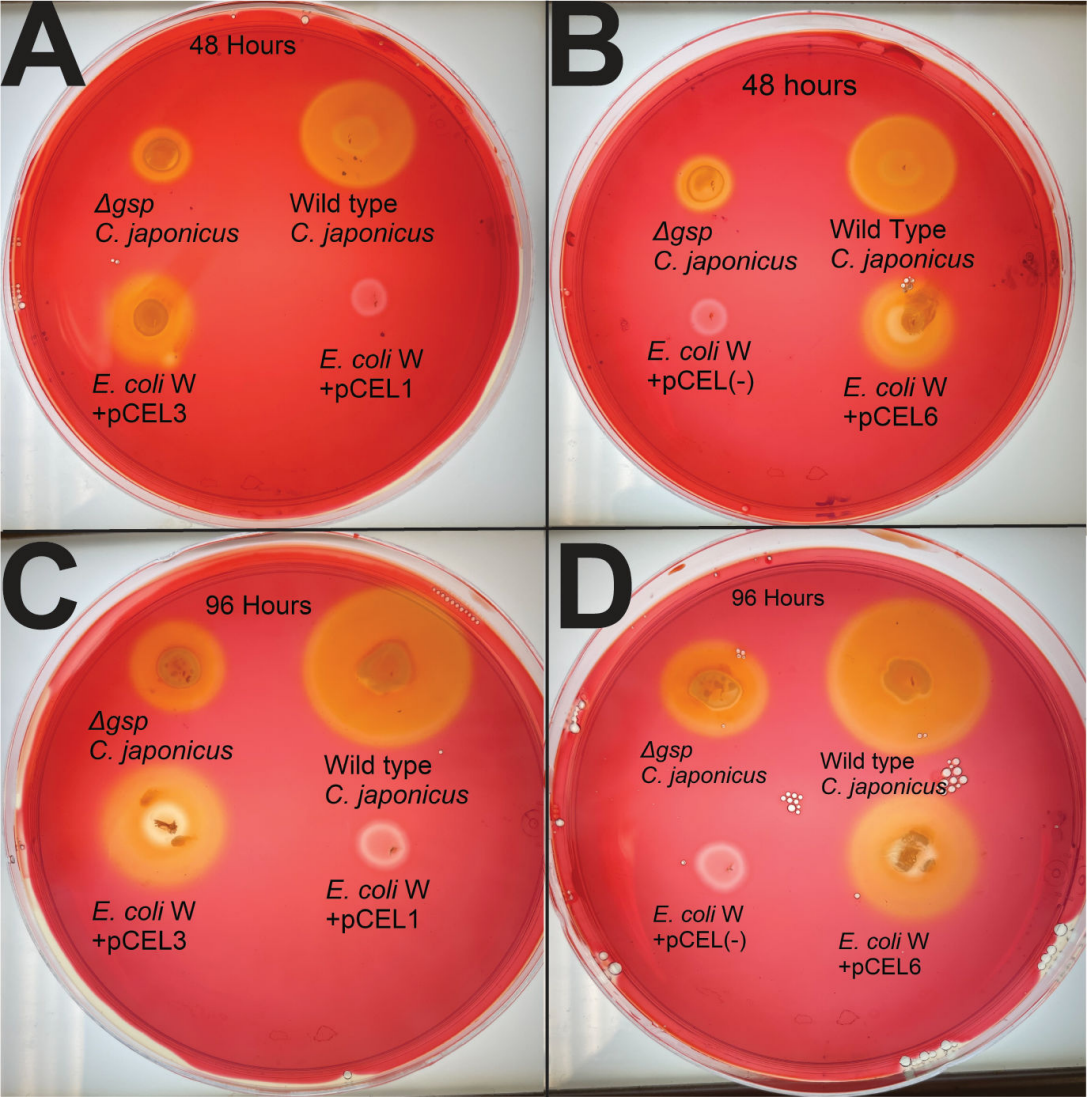
Congo Red staining of CMC agar plates with engineered *E. coli* W strains expressing *C. japonicus* genes that encode cellulases. Wild-type *C. japonicus* and a Δ*gsp* mutant are shown as positive and negative controls, respectively. (**A and B**) 48 hour incubation before staining. (**C and D**) 96 hour incubation before staining.

To determine if the long lag phase of the pCEL3 and pCEL6 strains when grown using CMC and the inability grow on filter paper was due to the heterologously expressed CAZymes not being exported or having poor activity, a Congo Red staining assay (43) was used to detect the secreted endocellulase activity. As shown in Fig. 4, *C. japonicus* wild-type and a Δ*gsp* mutant were used as positive and negative controls, respectively, to identify areas of CMC degradation. Wild-type *C. japonicus* had a large zone of clearing from the secreted cellulase activity, while the Δ*gsp* mutant had a very small zone of clearing caused by the cellulase activity from lysed cells. As expected, all zones of clearing were enlarged with increased incubation times (48 vs 96 hours). The VOC and pCEL1 strains of *E. coli* W had no zone of clearing at any time, while the pCEL3 and pCEL6 strains had distinct zones of clearing that increased from 48 to 96 hours, indicating that the *C. japonicus* genes were being expressed in *E. coli* W and that the encoded cellulases were active and nonspecifically exported out of the cell.

### Cellodextrin and glucose uptake by *C. japonicus* during cellulose degradation are efficient

**Fig 5:**
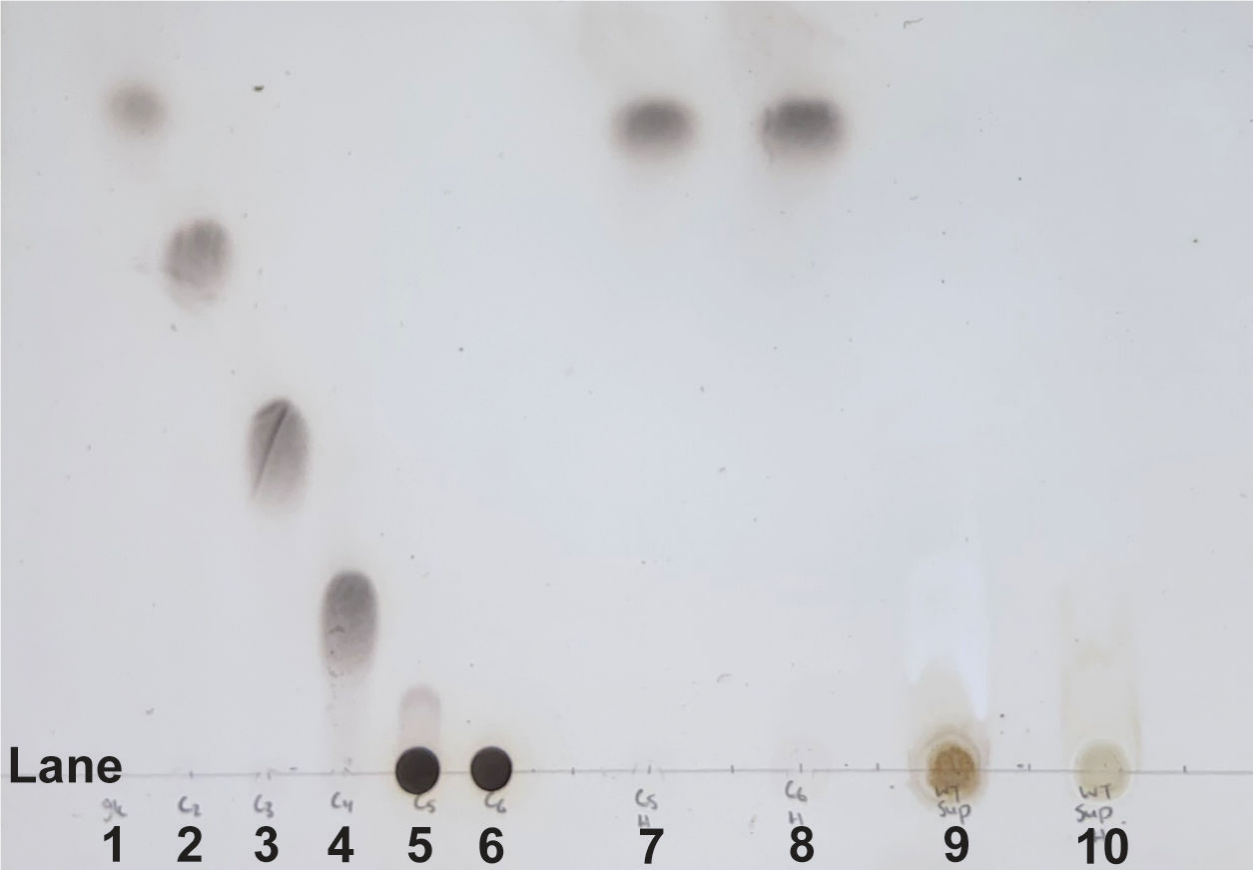
TLC analysis to identify glucose or cellodextrins released into the medium during *C. japonicus* growth using insoluble cellulose. Lanes 1–6 show standards that include glucose, cellobiose, cellotriose, cellotetrose, cellopentose, and cellohexaose, respectively. Lane 7 shows acid hydrolyzed cellopentose, and Lane 8 shows acid hydrolyzed cellohexaose. Lane 9 shows the supernatant from a T_120_ culture of *C. japonicus* that was treated with kanamycin at T_72_. Lane 10 shows an acid-hydrolyzed supernatant from a T_120_ culture of *C. japonicus* that was treated with kanamycin at T_72_.

A combination of thin layer chromatography (TLC) and an enzymatic glucose assay was used to determine what cellodextrins were being generated during *C. japonicus* insoluble cellulose degradation. A TLC solvent system previously published for starch oligosaccharides (40) was able to resolve glucose and cellodextrins with a degree of polymerization (DP) between 2 and 4. Larger soluble cellodextrins (cellopentose and cellohexaose) remained at the origin of application (Fig. 5). Acid hydrolysis of larger (DP >5) cellodextrin standards resulted in complete conversion to glucose that was detectable by TLC.

To identify the cellodextrins generated *in vivo*, a late exponentially growing culture of *C. japonicus* using filter paper was treated with kanamycin to kill the cells but allow cellulose degradation to continue. After a 48 hour incubation, where we observed no increase in OD_600_, supernatant samples were collected and filtered. Supernatant analysis by TLC did not detect any cellodextrins or glucose. There was a dark spot at the origin like that of cellohexaose. Acid hydrolysis of the filtered supernatant did not yield any detectable glucose or cellodextrins. A control experiment using serial dilutions of a 10 mM glucose standard determined that the detection limit for the TLC was approximately 0.2 µg glucose (Fig. S7).

**Fig 6:**
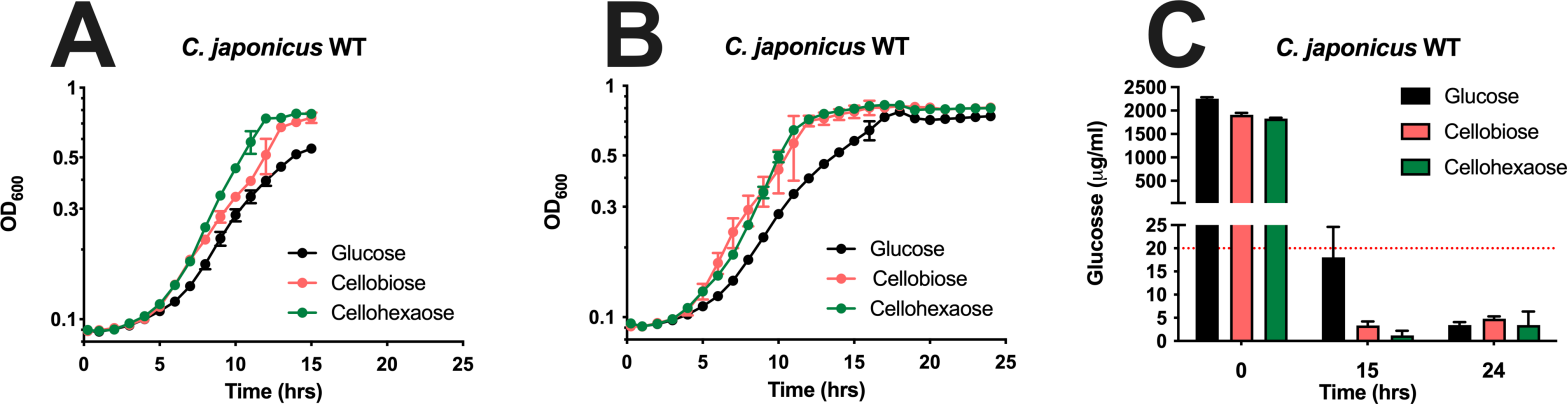
Growth and sugar assays to calculate the concentration of monosaccharide/oligosaccharide remaining in the medium after 15 (**A**) and 24 (**B**) hours of incubation. Wild-type *C. japonicus* cells were grown in the defined medium with either glucose, cellobiose, or cellohexaose as the sole carbon source. For cellobiose and cellohexaose, an exogenous β-glucosidase was added to convert all cellodextrin to glucose before quantitation. Starting sugar concentrations were also measured as controls (**C**). All oligosaccharides were reduced to glucose before quantification, with error bars representing standard deviation from biological triplicate experiments.

A previous study examined glucose and cellobiose uptake in *C. japonicus* and found that by 15 hours of growth, all glucose (16 mM starting concentration) had been consumed (22). During the same timeframe, a parallel culture of C. *japonicus* was able to consume approximately 90% in a cellobiose medium (18 mM starting concentration). To determine if larger cellodextrins are consumed at a similar rate, we grew *C. japonicus* using glucose, cellobiose, or cellohexaose, each with equivalent glucose starting molar concentration, as the sole carbon source for 15 or 24 hours and then removed biological triplicate samples. As shown in Fig. 6A and B, *C*. *japonicus* has a slightly faster growth rate on cellodextrins compared to glucose (e.g., 0.32 gen/hr compared to 0.23 gen/hr when using cellohexose). Samples taken from cellodextrin media were incubated with a β-glucosidase to cleave any remaining cellodextrins to glucose before performing a glucose detection assay. Our results for glucose and cellobiose were similar to those previously observed, specifically that by 15 hours the majority of substrate was consumed. While there was slightly more glucose at 15 hours compared to 24 hours, both values were below the limit of quantitation. Interestingly, we calculated that less than 0.2% cellohexaose remained in the medium after 15 or 24 hours (Fig. 6C).

**Fig 7:**
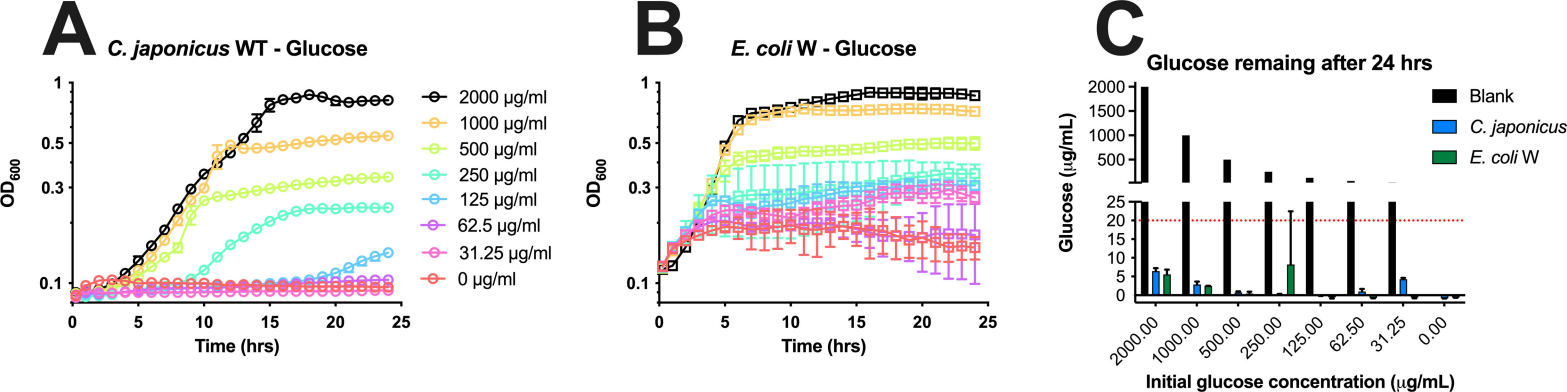
Growth analysis of *C. japonicus* and *E. coli* W with stepwise reduction in the glucose concentration and measurement of remaining glucose after 24 hours. (**A**) *C. japonicus* growth; (**B**) *E. coli* W growth. All growth experiments were performed in biological triplicate. Error bars represent standard deviation, but in many cases may be too small to be seen. (**C**) Glucose quantitation after 24 hours, with error bars representing standard deviation from biological triplicate experiments. The black bars represent calculated (not measured) initial glucose values.

To further test the glucose-scavenging capacity of *C. japonicus*, we performed growth experiments with stepwise reductions in glucose (Fig. 7A). As expected, with decreasing glucose concentration, there was a reduction in growth; however, a surprising result was that *C. japonicus* was unable to grow using 63 and 31 μg/mL glucose; however, after 24 hours, there was detectable but not quantifiable glucose (Fig. 7C). While the glucose consumption trends were similar for *E. coli* W, in contrast to *C. japonicus,* the *E. coli* W strain was able to grow using the lowest concentrations of glucose and had a similar initial growth rate for all glucose concentrations tested (Fig. 7B).

The final series of experiments specifically examined the D*cbp2D* or Δ*cbp2E* single-mutant strains when grown using cellodextrins to assess if the Cbp2D or Cbp2E proteins were important for insoluble cellulose only, or if they were also important for cellodextrin utilization. As expected, WT *C. japonicus* was able to grow using DP2-6 cello-oligosaccharides and the Δ*cbp2D* or Δ*cbp2E* single-mutant strains grew similarly to WT, both in terms of the growth rate and maximum growth achieved (Fig. S8).

## DISCUSSION

With the current study, we have provided further evidence for the importance of Cel5B and Cel6A in *C. japonicus* cellulose degradation, found negligible functional redundancy, highlighted the importance of two carbohydrate-binding proteins (Cbp2D and Cbp2E), and revised the model to include efficient cellodextrin and glucose transport as part of an environmental strategy to maximize the energetic economics for bacterial cellulose utilization.

### *C. japonicus* CAZyme-encoding gene expression varies little during deconstruction of crystalline insoluble cellulose regardless of the growth rate

A prior transcriptomic report for *C. japonicus* grown using microcrystalline Avicel observed biphasic growth and numerous changes in CAZyme-encoding gene expression between exponential-growth and stationary-phase cells (21). Avicel is an insoluble cellulose substrate with 60%–70% crystalline and 30%–40% amorphous regions (51), which the authors hypothesize was the reason for the observed *C. japonicus* growth dynamics. *C. japonicus* likely degraded the amorphous areas prior to more recalcitrant regions, which is supported by the higher efficacy of CAZymes on non-crystalline regions of cellulose (21). To ascertain gene expression patterns that more accurately reflect environmentally and industrially relevant types of cellulose, we performed an RNA-seq analysis using filter paper, which is substantially more crystalline than Avicel (51). An additional rationale for using filter paper instead of Avicel was that this substrate has a reduced weight-to-surface area ratio that is more representative of ecological conditions and presents a greater challenge for enzymatic degradation.

There were two major observations from the *C. japonicus* RNA-seq analysis. The first was the strong evidence for a substrate-sensing mode of gene regulation. For both the exponential- and stationary-phase comparisons for glucose versus cellulose-grown cells, there were several significantly upregulated CAZyme-encoding genes, including *cel5B*, *cel6A*, *lpmo10B*, *cbp2D*, and *cbp2E* (Tables S2 and S3). Furthermore, MA plot analysis indicated that these genes were not only differentially expressed compared to glucose but were also highly expressed and provided further evidence that *cel3B* is constitutively expressed at a high level (Fig. 1). What was striking was that there were no significantly upregulated CAZyme-encoding genes when we compared exponential- versus stationary-phase cellulose-grown cells. We hypothesize this is due to the highly recalcitrant nature of filter paper and the requirement of the same suite of cellulases required to deconstruct it, regardless of growth rate. Specifically examining CAZyme-encoding genes, *C. japonicus* has eight genes predicted to encode GH5 enzymes and three genes predicted to encode GH9 enzymes; however, only *cel5B*, *cel5E*, and *cel5G* were significantly upregulated under cellulose conditions. Comprehensive mutational analyses found that only the Δ*cel5B* mutant elicited an observable growth defect (Fig. 2); however, it should be noted that this strain was still able to achieve a WT level of maximum growth. This result suggests that other endoglucanases (possibly from the GH5 family) can compensate for the loss of Cel5B. An alternative explanation is that the proteins that we categorized as the minimal core set can still degrade cellulose, albeit at a reduced rate in the absence of Cel5B. Future work to parse these two options could include the construction of strains that have multiple genes that encode GH5 and/or GH9 enzymes in *C. japonicus* or RNA-seq analysis of the Δ*cel5B* mutant to identify these compensating enzymes and determine if the cellulose-detection ability of *C. japonicus* is significantly altered if the major endoglucanase is absent. Other bacteria have been reported to regulate gene expression via substrate sensing, with one example being *Fibrobacter succinogenes,* where collection of CAZyme-encoding genes was significantly upregulated when cells were grown using insoluble cellulose compared to cellobiose or glucose (52). However, the ability of *C. japonicus* and *F. succinogenes* to sense cellulose is likely different from other bacteria proficient at cellulose deconstruction. Two reports using *Clostridium thermocellum* found that CAZyme-encoding gene expression was dependent on the growth rate (53) (54), and a later study using *C. thermocellum* grown using lignocellulosic biomass found that cellulosome protein-encoding genes were significantly upregulated using recalcitrant biomass compared to cellobiose independent of the growth rate (55).

The second notable, and unexpected, observation was the large number of nutrient transporters upregulated during cellulose-dependent growth. The upregulation of multiple ABC transporters and components of TonB-dependent transports (TBDTs) suggests that *C. japonicus* uses these mechanisms to import cellodextrins (Tables S2 and S3). TBDTs have increasingly been shown as important to bacterial oligosaccharide import, and the types of sugar substrates that are recognized by TBDTs have yet to be exhausted (56). One example found in *Bacteroides thetaiotaomicron* found that SusCD proteins are important for starch oligosaccharide import, but with over 100 predicted TBDTs in this bacterium, there are likely many more substrates to be found (57). For example, there is emerging work that the uptake of levans, a polysaccharide class comprised β−2,6 fructose that shows promise as a prebiotic, by *B. thetaiotaomicron* is also mediated by TBDTs (58–60). Future work from our group aims to identify and characterize the TBDTs from *C. japonicus,* with an emphasis on those that import cellodextrins and other oligosaccharides.

### GH5 functional redundancy is minimal while some carbohydrate-binding proteins have increased importance in *C. japonicus* cellulose deconstruction

The *C. japonicus* genome is predicted to contain over 200 genes that encode carbohydrate-active enzymes, which includes the eight from the GH5 family (14). While CAZyme functional redundancy has been proposed as a feature of microbial communities (61), it is still unclear to what extent CAZymes are redundant within an individual bacterium, but a review of cellulase distribution across taxa suggests that it is likely low (62). Specific to this study, several previous papers suggest that the phenomenon is not present in *C. japonicus* (22, 63, 64); therefore, to better assess which GH5 and GH9 cellulases are required versus accessory, we performed a comprehensive mutational analysis.

Our data suggest little functional redundancy for cellulose deconstruction is found in *C. japonicus*. Growth defects were observed for the sole GH6-encoding gene (*cel6A*) and only for two of the GH5-encoding genes (*cel5A* and *cel5B*). None of the three predicted GH9-encoding or GH45-encoding genes grew differently than wild-type (Fig. 2). It was not surprising that only a few GH5-encoding genes were important for cellulose degradation given that a previous report identified three (*cel5D*, *cel5E*, and *cel5F*) encoded highly active endoxyloglucanases (41). Similarly, GH9 CAZymes have been shown active on β-glucan (65) (66), and ongoing work by our group is using a broad array of glycan substrates to determine the physiologically relevant functions of the uncharacterized *C. japonicus* GH5 and GH9 enzymes.

The current model for cellulose utilization in *C. japonicus* describes the Cbp2D and Cbp2E proteins as accessory to Lpmo10B, with putative roles as electron donors (21). Interestingly, single-gene deletion of *cbp2D* or *cbp2E* had poorer growth than a single-gene deletion mutant of *lpmo10B* in that study. We hypothesize that the absence of a growth defect for the *lpmo10B* mutant is due to the Cbp2D and Cbp2E proteins also being able to act on insoluble crystalline cellulose and being more potent enzymes than Lpmo10B. Lytic polysaccharide monooxygenases (LPMOs) utilize oxidation to break glycosidic bonds where electrons are required to activate molecular oxygen and cleave the glycosidic linkage (67, 68). Electrons can also be provided by small reducing molecules such as ascorbic acid (67) (69) or enzymatically by proteins such as cellobiose dehydrogenases (70). *C. japonicus* does not possess a cellobiose dehydrogenase, but Cbp2D contains a fibronectin type III domain, and this type of domain was shown in *C. thermocellum* to both assist in protein-protein interactions (71) and improve the hydrolysis of cellulose by GH9 CAZymes (72). More recently, the X183 domain of Cbp2D was shown to be a *c*-type cytochrome that, in isolation, could initiate Lpmo10B copper reduction to activate cellulose oxidative cleavage (23). Along with the presence of two additional domains with no known role (X158 and X132), it is possible that Cbp2D may perform more than one cellulose degradation function. A surprising result during the growth analysis was that the *cbp2E* mutant strain grew more poorly using filter paper compared to the *lpmo10B* mutant (Fig. 2D). Comparing the protein sequence of Cbp2D and Cbp2E, the former is 1,122 amino acids and the latter is 350 amino acids. Predicted structures from AlphaFold (via the UniProt database; ID#s: B3PLJ5 and B3PLJ6) suggest the X183 domain protrudes from the rest of the Cbp2D protein and is structurally distant from the CBM2 domain (73, 74). The AlphaFold structure of Cbp2E indicates two domains, one of which is predicted to be a CBM2, connected by a long tether (Fig. S9). Our current hypothesis is that Cbp2D and Cbp2E are working as a heterodimer, which would account for the similar growth phenotypes when either is deleted, and this complex is directly cutting cellulose by a novel mechanism but may also provide the redox power for Lpmo10B function. Ongoing work by our group will continue mutational analysis of the *cbp2D* and *cpb2E* genes to further characterize their roles in insoluble cellulose deconstruction.

The results in this report have led us to predict that other Cbps from *C. japonicus* may have novel mechanisms of polysaccharide degradation. Current gene annotation lists 18 Cbps, specifically six from the CBM2 family (which includes Cbp2D and Cbp2E), three from the CBM6 family, one from the CBM10 family, one from the CBM26 family, two from the CBM32 family, two from the CBM33 family, and three from the CBM35 family (Table S5). The CBM33 proteins in *C. japonicus* have been re-annotated as Lpmo10A and Lpmo10B, and more generally, the CBM33 family has been retired (21, 64, 75). Given that CBM6, CBM10, CBM26, and CBM35 have known roles in the binding of xyloglucan, cellulose/xylan mixtures, starch, or mannans, respectively (76), future work in *C. japonicus* will include a comprehensive mutational analysis of *cbp* genes to provide insights into their physiological functions in the context of polysaccharide utilization. For example, previous RNA-seq experiments in *C. japonicus* have identified *cbp26A* as significantly upregulated when grown using plant mannans, xyloglucan, or starch compared to glucose, and similar comparative analyses will help prioritize gene targets in subsequent mutational studies (34, 35, 63).

### The essential cellulases for *C. japonicus* cellulose utilization are also minimally sufficient to enable robust cellodextrin and soluble cellulose growth in *E. coli*

Substantial previous work has attempted to engineer *E. coli* strains to produce CAZymes, with the goal of many studies being the production of a consolidated bioprocessor, which has been met with limited success. For example, a previous study over-expressed the *C. japonicus cel3A* and *cel3B* genes in *E. coli* MG1655, which resulted in negligible growth on insoluble cellulose even when coupled with cellulases from *Bacillus sp*. D04 (77). These lackluster results are unsurprising given the numerous challenges of heterologously expressing genes in *E. coli*, some of which include strain choice, gene expression, protein activity, and protein trafficking. Our work sought to build from previous attempts and produce an engineered strain of *E. coli* with improved cellulose utilization capabilities.

In terms of strain selection, *E. coli* K-12, which contains a silenced *bgl* operon (78), was deemed an unsuitable chassis; however, *E. coli* W has limited capacity to use cellobiose and can harbor large plasmids. Growth of the engineered strain using cellodextrins indicated that expression from pCEL plasmids resulted in active *C. japonicus* enzymes (Fig. 3); however, it is unclear if the physiologically relevant cellulose cleavage is internal or external in the engineered strain. We hypothesize that the failure of the *E. coli* W/pCEL strains to grow using filter paper is likely due to the poor activity of Lpmo10B and/or Cbp2D/E. The addition of glutathione as an electron donor for Lpmo10B did not enable the *E. coli* W/pCEL6 strain to grow, and we were unable to test ascorbate as an alternative electron donor because *E. coli* W can natively use it as a carbon source (48), which meant that the LPMO was reliant on any redox power provided by Cbp2D/E. Furthermore, given the large size of Cbp2D, it is unclear how much of the protein was exported, and future experiments using either secretomics or Western blotting could address this uncertainty. The Congo Red plate assays indicated that Cel5B was exported and allowed for degradation of CMC (Fig. 4), which suggests that at least some heterologously expressed proteins were released outside of the cell. The export of the *C. japonicus* proteins was nonspecific in the engineered strain and possible due to limited cell leakage or lysis, so future work will use fusions to OsmY or YebF to increase the amount of heterologously expressed protein that is actively secreted, which has been done in other systems (79). Alternatively, an exclusively *in vitro* approach using purified enzymes might also yield some insights regarding how combinations of the identified minimal CAZyme set deconstruct cellulose types of varying solubility.

### Both short-chain and larger cellodextrins are consumed by *C. japonicus* at a similar rate

Thin-layer chromatography of acid-hydrolyzed insoluble cellulose medium yielded no detectable cellodextrins or glucose, which suggested both short-chain and long-chain oligosaccharides were imported into *C. japonicus* rapidly during cellulose degradation (Fig. 5). A previous study had noted that cellobiose consumption by *C. japonicus* was highly efficient (22); however, the consumption rate of larger cellodextrins remained unclear. An assay to measure cellodextrin uptake by *C. japonicus* found that the bacterium was able to consume 99% of cellobiose and cellohexaose after 15 hours (Fig. 6), indicating that the consumption rate of the two cellodextrins was similar. An independent glucose uptake assay to assess *C. japonicus* growth on decreasing glucose concentrations found that efficient glucose uptake was observed with higher glucose concentration, but surprisingly also with low glucose concentrations, even though those lower glucose concentrations did not support growth (Fig. 7B). These data provide further evidence that *C. japonicus* is a polysaccharide utilization specialist and has a growth preference for oligosaccharides over glucose, which contrasts with the *E. coli* W strain that was able to grow using low levels of glucose.

### A revised model for cellulose utilization by *C. japonicus* predicts efficient uptake of cellodextrins and direct polymer cleavage by a Cbp2D/E heterodimer

**Fig 8:**
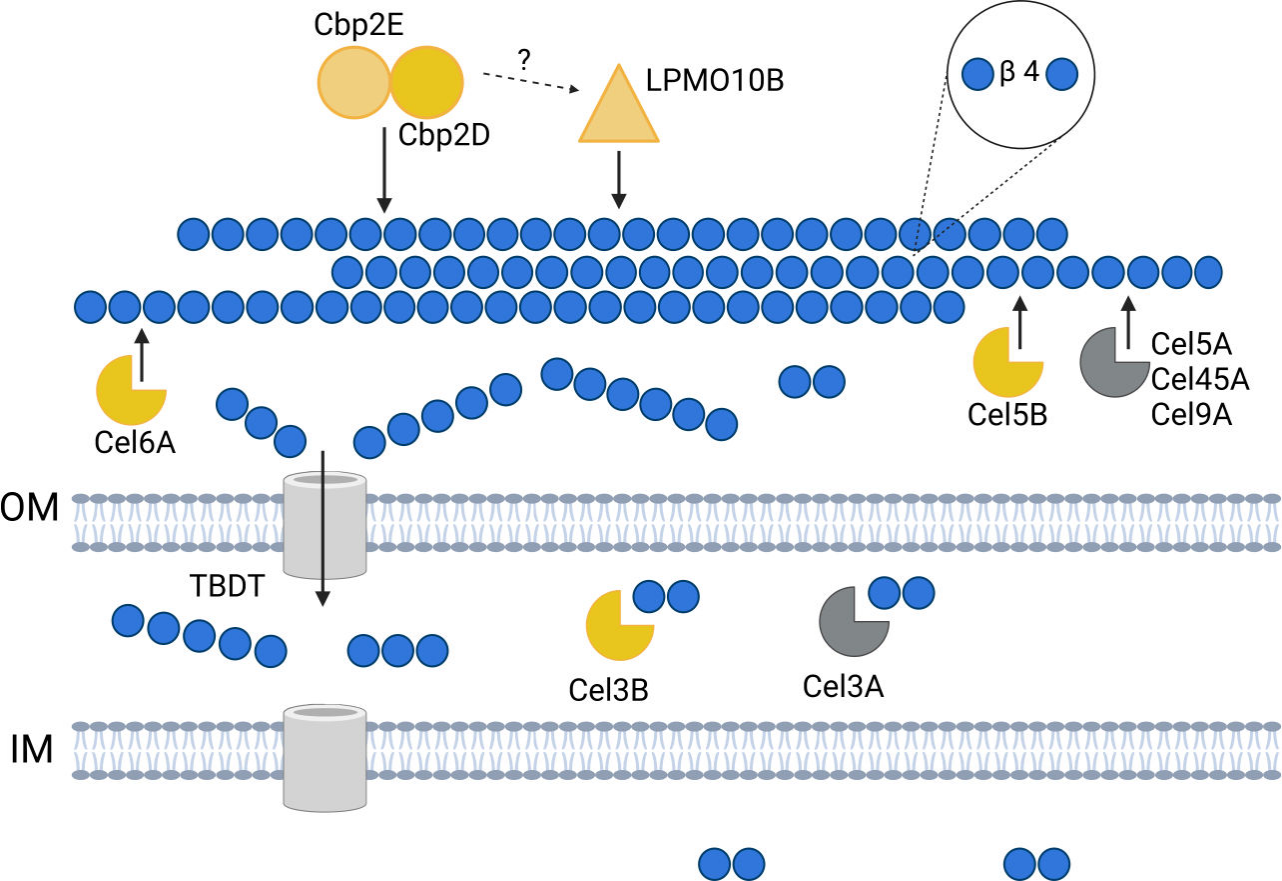
Revised model for cellulose deconstruction by *C. japonicus*. The six enzymes that are essential for cellulose deconstruction are highlighted in yellow, while those in gray have been shown to play secondary roles. The gray tubes in the membranes represent the yet-to-be-identified transporters that are specific for cello-oligosaccharides. Finally, the roles of the Cbp2D and Cbp2E proteins have been updated to include an activity that directly cuts cellulose polymers in addition to the predicted activity of electron transfer to Lpmo10B. This figure was produced using assets from BioRender.

The existing model for *C. japonicus* cellulose bioconversion emphasizes the importance of the Lpmo10B and Cel5B enzymes, with supporting roles for Cel6A and Cel3B (19). This report refines that model by expanding the view of cellulose utilization in *C. japonicus* at the initial and final stages, specifically the degradation of recalcitrant insoluble cellulose and the import of soluble cellodextrins (Fig. 8). Based on the strength of the *C. japonicus* gene deletion experiments, specifically those with the single and double mutants, we hypothesize that Cbp2D and Cbp2E are working together as a heterodimer in a manner that goes beyond electron transfer to the LPMO. Future *in vitro* enzymatic or structural studies with the Cbp2D and Cbp2E proteins would provide additional evidence for a potentially novel mechanism of insoluble cellulose cleavage in bacteria.

The second, and perhaps more substantial, revision is the inclusion of an efficient mechanism of cellodextrin import after cellulose degradation. We hypothesize that the absence of detectable cellodextrins during cellulose degradation is due to rapid oligosaccharide uptake via TonB-dependent transporters (TBDTs). A previous study examined the abundance of TBDTs over 226 species and proposed that bacteria with an overabundance of TBDTs are robust polysaccharide scavengers (80). *C. japonicus* has 75 genes predicted encoding TBDT components, and two have been characterized due to their importance in chitin oligosaccharide metabolism (81). The use of TBDTs has been proposed to be part of a “selfish mechanism” to maximize nutrient recovery by CAZyme-secreting bacteria, specifically *Bacteroides thetaiotaomicron* (57, 82). Recent studies have suggested that extracellular hydrolysis is mutually exclusive from selfish uptake of oligosaccharides (83) (84); however, these studies focus on marine consortia where the diffusion of secreted enzymes is likely much more pronounced than in terrestrial environments. Consequently, ongoing work by our group is exploring the dynamic between shared goods (e.g., secreted CAZymes by *C. japonicus*) and cellodextrin uptake mechanisms to maximize the energetic return during polysaccharide degradation in an environment. The energetic economics of bacterial recalcitrant polysaccharide utilization was recently reviewed (18), and advances in that area will be broadly applicable to better understanding how microbial communities cooperate or compete in natural environments.

## Data Availability

All cellulose RNAseq data generated during the study were submitted to NCBI GEO under accession number GSE287208.

## SUPPLEMENTAL MATERIAL

File S1 aem.00818-25-s0001.pdf Figures S1 to S9; Tables S1, S4, and S5.

File S2 aem.00818-25-s0002.xlsx Tables S2 and S3.

ASM does not own the copyrights to Supplemental Material that may be linked to, or accessed through, an article. The authors have granted ASM a non-exclusive, world-wide license to publish the Supplemental Material files. Please contact the corresponding author directly for reuse.
