# Supplementary material for: The physiological functions of the Cbp2D and Cbp2E proteins are important for insoluble cellulose-dependent growth in Cellvibrio japonicus
Source: Appl Environ Microbiol. 2025 Sep 4;91(10):e00818-25. doi: 10.1128/aem.00818-25 (PMC12542752; doi:10.1128/aem.00818-25)
Supplement: File S1 — Figures S1 to S9; Tables S1, S4, and S5. [file aem.00818-25-s0001.pdf]

The physiological functions of the Cbp2D and Cbp2E proteins are important for cellulose-dependent growth in *Cellvibrio japonicus*

Baily E. Kakacek*, Jiabao Liang*, Kyle A. Dickerson, and Jeffrey G. Gardner[#]

Running Title
Cellulose utilization in *C. japonicus*

Keywords
CAZyme, cellodextrin, cellulase, cellulose, *Cellvibrio japonicus*, polysaccharide degradation

Author Affiliations
Department of Biological Sciences, University of Maryland - Baltimore County
Baltimore, Maryland, USA

*These authors contributed equally to the manuscript. Author order was determined alphabetically by last name

[#]Correspondence
Jeffrey G. Gardner
Department of Biological Sciences
University of Maryland - Baltimore County
Email: jgardner@umbc.edu
Phone: 410-455-3613
Fax: 410-455-3875

SUPPLEMENTAL FIGURES

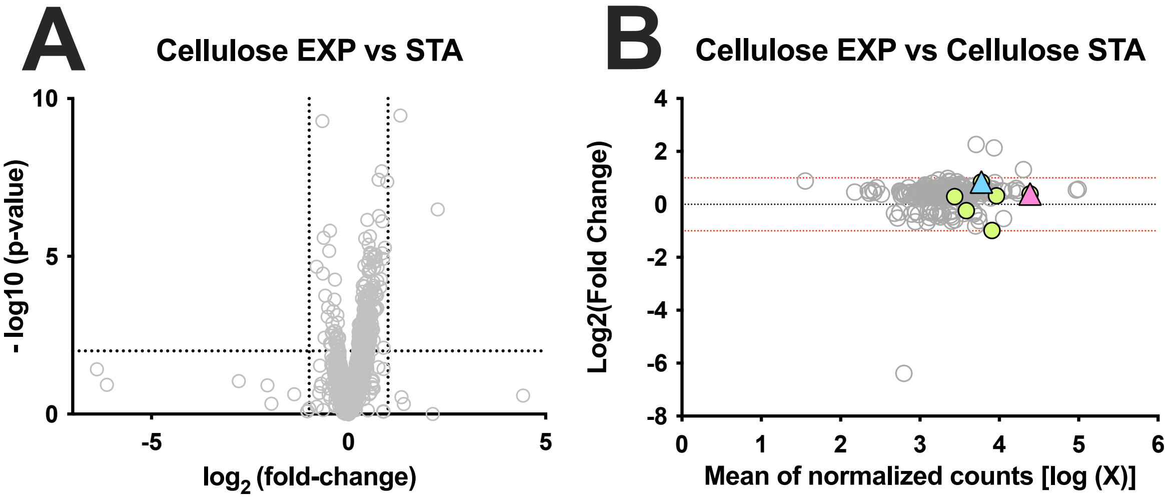

Figure S1. There were no CAZyme-encoding genes or TBDT-encoding genes significantly up-regulated during stationary phase on cellulose compared to exponential growth on cellulose, and only two genes up-regulated overall (CJA_2120 and CJA_2729) (**A**). Differential gene expression of *C. japonicus* cells grown on cellulose during exponential growth compared to stationary phase is shown in the volcano plot. Fold-change (log_2 adjusted) is shown on the X-axis and *p*-value ($-log_{10}$ adjusted) is on the Y-axis. Dotted lines represent significant cutoffs for fold-change and *p*-value (fold-change $\geq$ 2 and p-value $\leq$ 0.01). Each grey circle represents a *C. japonicus* gene. The MA plot displays differential expression data of wild-type *C. japonicus* grown using either glucose or filter paper as the sole carbon source. Fold change (log_2) is on the Y-axis and mean of normalized counts (log) is on the X-axis. Genes with a *p*-value of ≥ 0.01 are excluded from the plot. Dashed lines depict log_2 fold-change thresholds of -1 and 1. (**B**) Cells using insoluble cellulose, exponential phase versus stationary phase. Each open circle represents a *C. japonicus* gene and each green closed circle represents a CAZyme-encoding gene. The blue triangle represented the *cel5B* gene and the pink triangle represents the *cel3B* gene.

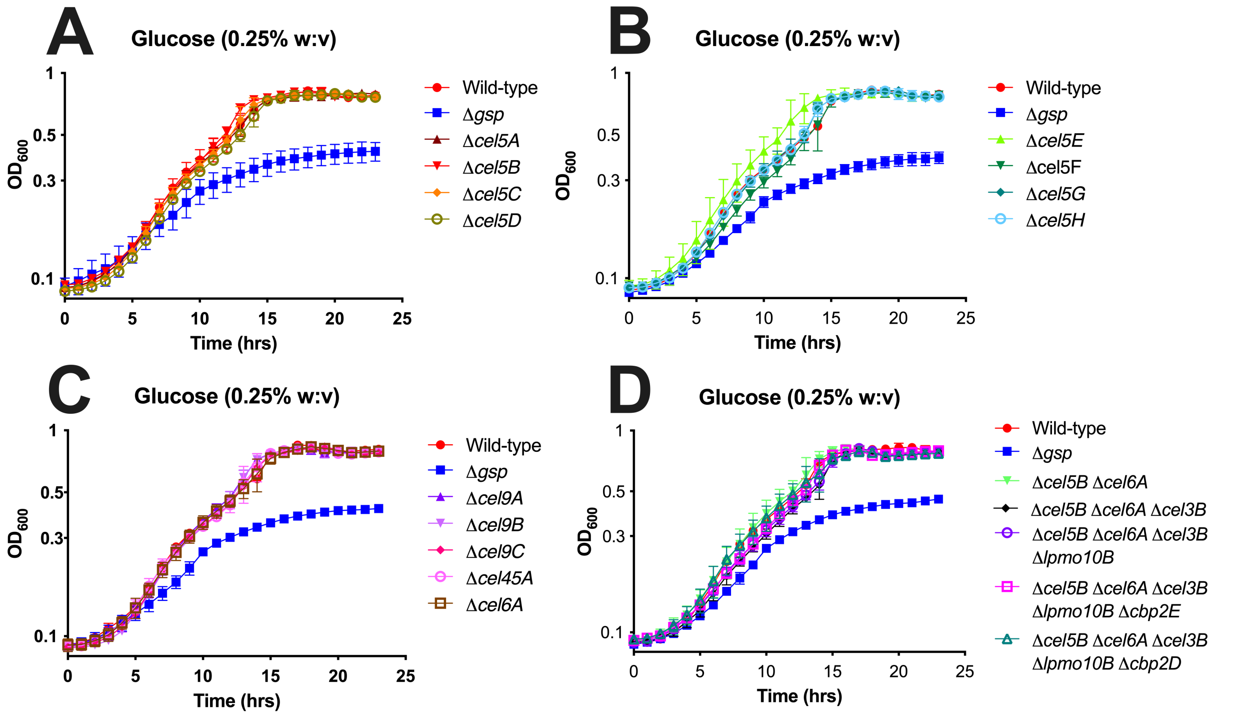

Figure S2. All *C. japonicus* single deletions for genes that encode cellulases all grow like wild-type when using glucose as the sole carbon source in minimal media. (**A**) Gene deletion mutants for *cel5A-D*, (**B**) Gene deletion mutants for *cel5E-H*, (**C**) Gene deletion mutants for *cel9A-C*, *cel45A*, *cel6A*, and (**D**) multi-gene mutants. In all panels, wild-type and a Δ*gsp* mutant are show as positive and negative controls, respectively. All growth experiments were performed in biological triplicate. Error bars represent standard deviation, but in many cases may be too small to be seen.

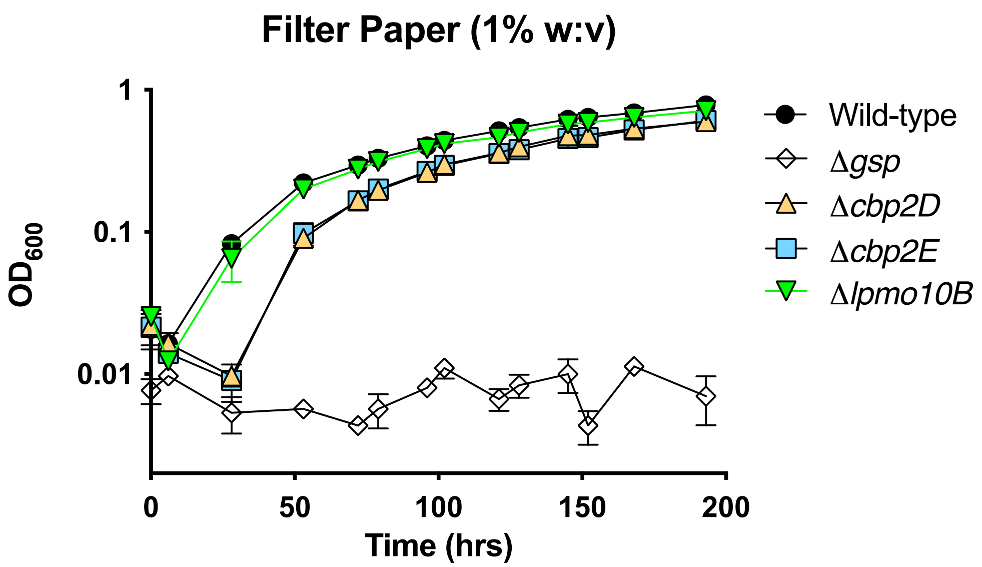

Figure S3. *C. japonicus* wild-type and a Δ*lpmo10B* single mutant strain grow similarly using insoluble cellulose (filter paper) as the sole carbon source, while a Δ*cbp2D* or Δ*cbp2E* single mutant have a reproducible growth defect in the form a pronounced lag phase. The Δ*gsp* strain is included as a negative control. All growth experiments were performed in biological triplicate. Error bars represent standard deviation, but in many cases may be too small to be seen.

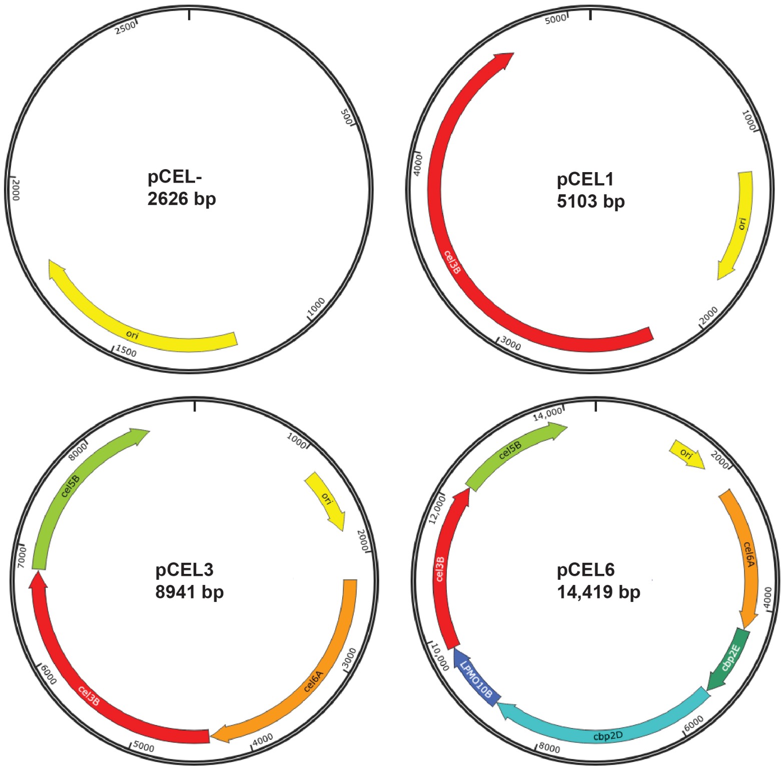

Figure S4. Plasmid maps for the pCEL series of vectors used to heterologously express genes that encode *C. japonicus* cellulases in *E. coli*. A kanamycin resistance cassette is present on all plasmids but is not shown. All plasmids have a pMB1 origin of replication and are derivatives of pUC-GW-Kan. Plasmid maps were made using SnapGene software.

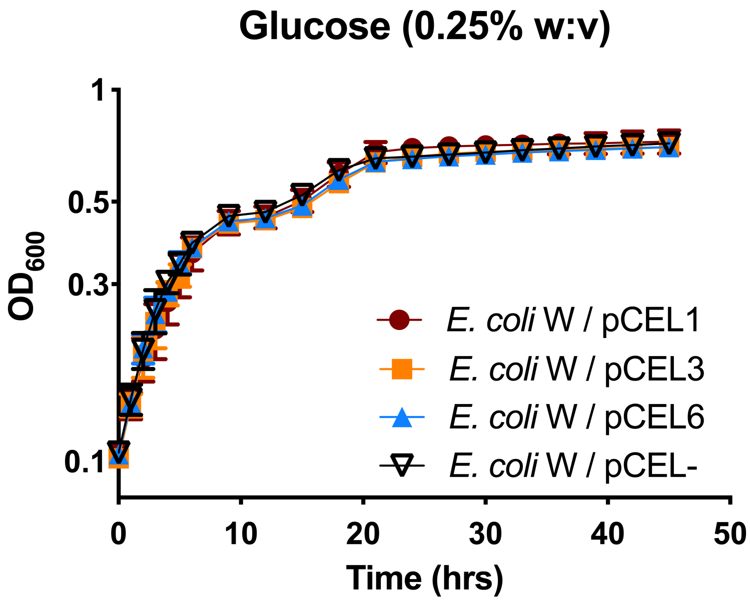

Figure S5. *E. coli* W strains containing the pCEL series of plasmids grow similarly when using glucose as the sole carbon source in minimal media. All growth experiments were performed in biological triplicate. Error bars represent standard deviation, but in many cases may be too small to be seen.

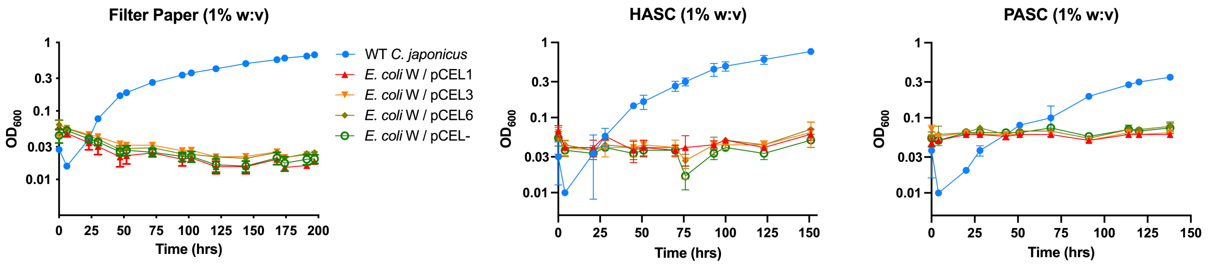

Figure S6. *E. coli* W strains containing the pCEL series of plasmids are unable to grow using insoluble cellulose as the sole carbon source in minimal media. Substrates tested included (**A**) filter paper, (**B**) hydrochloric acid swollen cellulose (HASC), and (**C**) phosphoric acid swollen cellulose (PASC). In all panels wild-type *C. japonicus* was used as a positive control. Error bars represent standard deviation from biological triplicate measurements, but in many cases may be too small to be seen.

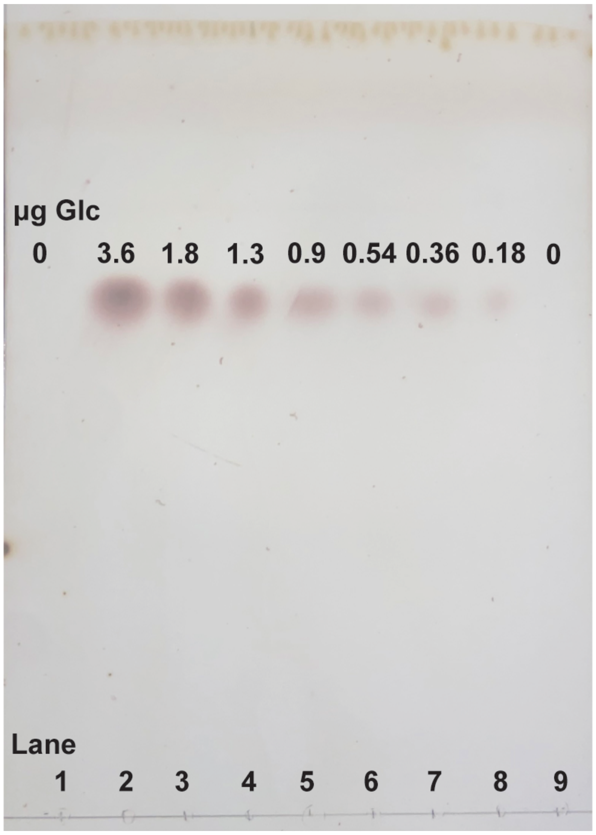

Figure S7. Thin layer chromatography (TLC) with increasing dilutions of glucose to identify the lowest visualizable concentration. Lane 1: Blank; Lane 2: 3.6 μg glucose; Lane 3: 1.8 μg glucose; Lane 4: 1.3 μg glucose; Lane 5: 0.9 μg glucose; Lane 6: 0.54 μg glucose; Lane 7: 0.36 μg glucose; Lane 8: 0.18 μg glucose; Lane 9: 0 μg glucose.

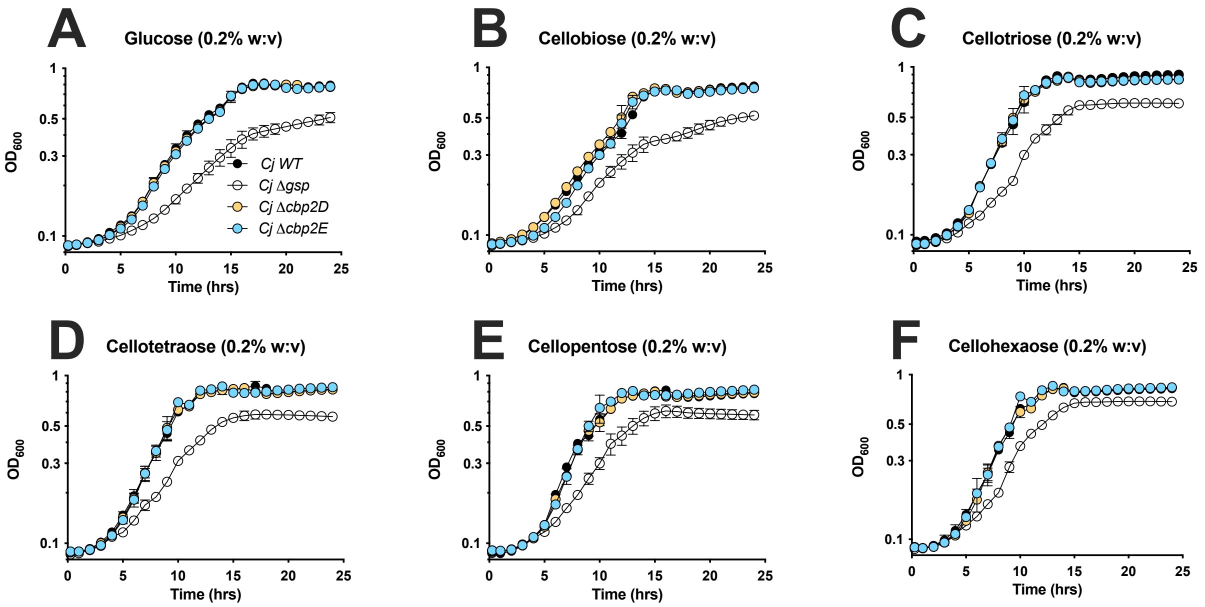

Figure S8. *C. japonicus* wild-type and the Δ*cbp2D* or Δ*cbp2E* single mutants grow similarly using cellodextrins as sole carbon sources. The Δ*gsp* mutant is shown to illustrate that secreted CAZymes are not essential for growth on cellodextrins. Glucose (**A**) was used a control substrate, while DP2-6 cellodextrins (**B – F**) represent all possible soluble cello-oligosaccharides. All growth experiments were performed in biological triplicate. Error bars represent standard deviation, but in many cases may be too small to be seen. It should be noted that the data shown in panel **B** was collected from a growth experiment that was independent from panels **A** and **C – F** (*i.e.* different microtiter assay plate) and is being shown here to facilitate interpretation.

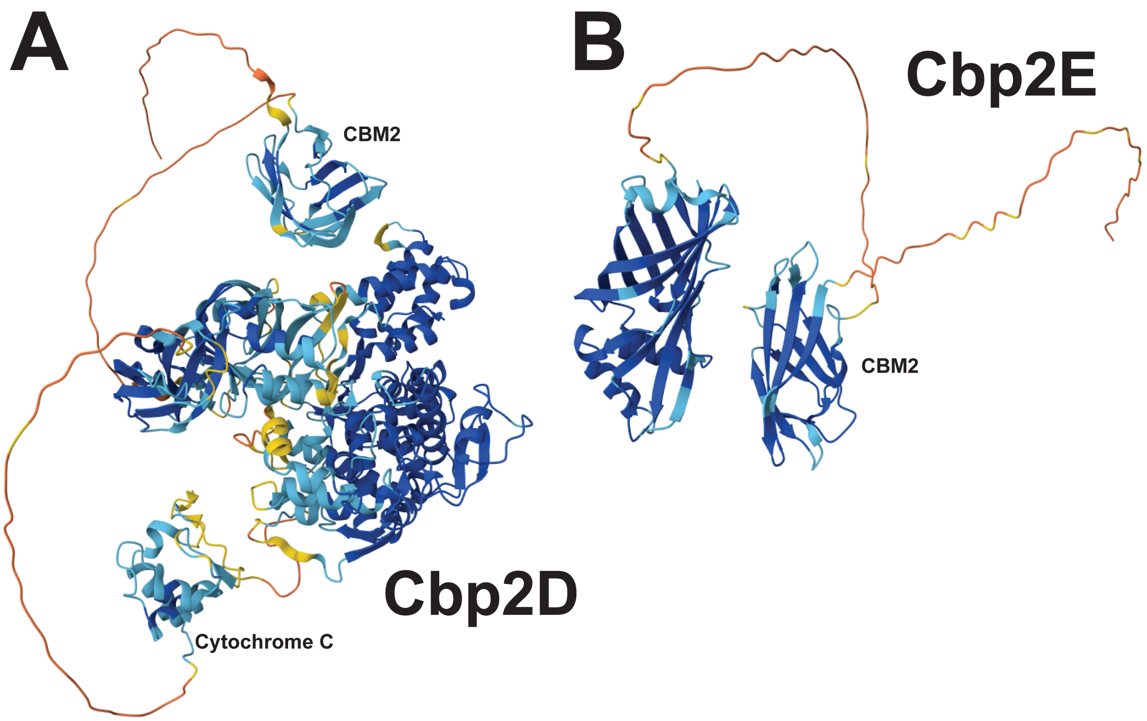

Figure S9. AlphaFold models of (**A**) Cbp2D and (**B**) Cbp2E.

SUPPLEMENTAL TABLES

Table S1A. Strains used in this study.

Strain Name	Genotype	Source
C. japonicus Ueda107	Prototroph (wild-type)	Laboratory collection
C. japonicus Δ*gsp* (CJA_3325-3333)	*C. japonicus Udea107* Δ*gsp*	Laboratory collection
C. japonicus Δ*cel5A* (CJA_1462)	*C. japonicus Udea107* Δ*cel5A*	This study
C. japonicus Δ*cel5B* (CJA_2983)	*C. japonicus Udea107* Δ*cel5B*	Laboratory collection
C. japonicus Δ*cel5C* (CJA_3369)	*C. japonicus Udea107* Δ*cel5C*	This study
C. japonicus Δ*cel5D* (CJA_3010)	*C. japonicus Udea107* Δ*cel5D*	Laboratory collection
C. japonicus Δ*cel5E* (CJA_3337)	*C. japonicus Udea107* Δ*cel5E*	Laboratory collection
C. japonicus Δ*cel5F* (CJA_2959)	*C. japonicus Udea107* Δ*cel5F*	Laboratory collection
C. japonicus Δ*cel5G* (CJA_2760)	*C. japonicus Udea107* Δ*cel5G*	This study
C. japonicus Δ*cel5H* (CJA_0619)	*C. japonicus Udea107* Δ*cel5H*	Laboratory collection
C. japonicus Δ*cel9A* (CJA_2472)	*C. japonicus Udea107* Δ*cel9A*	Laboratory collection
C. japonicus Δ*cel9B* (CJA_1633)	*C. japonicus Udea107* Δ*cel9B*	Laboratory collection
C. japonicus Δ*cel9C* (CJA_3804)	*C. japonicus Udea107* Δ*cel9C*	This study
C. japonicus Δ*cel45A* (CJA_0374)	*C. japonicus Udea107* Δ*cel45A*	Laboratory collection
C. japonicus Δ*cel6A* (CJA_2473)	*C. japonicus Udea107* Δ*cel6A*	Laboratory collection
C. japonicus Δ*cel5B* Δ*cel6A*	*C. japonicus Udea107* Δ*cel5B* Δ*cel6A*	Laboratory collection
C. japonicus Δ*cel5B* Δ*cel6A* Δ*cel3B*	*C. japonicus Udea107* Δ*cel5B* Δ*cel6A* Δ*cel3B*	Laboratory collection
C. japonicus Δ*cel5B* Δ*cel6A* Δ*cel3B* Δ*lpmo10B*	*C. japonicus Udea107* Δ*cel5B* Δ*cel6A* Δ*cel3B* Δ*lpmo10B*	Laboratory collection
C. japonicus Δ*cel5B* Δ*cel6A* Δ*cel3B* Δ*lpmo10B* Δ*cbp2D*	*C. japonicus Udea107* Δ*cel5B* Δ*cel6A* Δ*cel3B* Δ*lpmo10B* Δ*cbp2D*	This study
C. japonicus Δ*cel5B* Δ*cel6A* Δ*cel3B* Δ*lpmo10B* Δ*cbp2E*	*C. japonicus Udea107* Δ*cel5B* Δ*cel6A* Δ*cel3B* Δ*lpmo10B* Δ*cbp2E*	This study
E. coli DH5α	λ^- ϕ80d*lacZ*ΔM15 Δ(*lacZYA-argF*) *U169 recA1 endA1 hsdR17*(r_K^- m_K^-) *supE44 thi-1 gyrA relA1*	Laboratory collection
E. coli S17 λpir	Tp^r Sm^r *recA thi pro hsdR hsdM*$^+$	Laboratory

	RP4-2-Tc::Mu::Km Tn*7* λ*pir*	collection
E. coli MG1655 / pRK2013	F-, λ^-, *rph-1* pRK2013	Laboratory collection
E. coli W	*bgl* unsilenced	ATCC 9637
E. coli W / pCEL-	*bgl* unsilenced; pCEL-	This study
E. coli W / pCEL1	*bgl* unsilenced; pCEL1	This study
E. coli W / pCEL3	*bgl* unsilenced; pCEL3	This study
E. coli W / pCEL6	*bgl* unsilenced; pCEL6	This study

Table S1B. Plasmids used in this study.

Plasmid	Genotype	Source
pK18*mobsacB*	pMB1, *mob*$^{+}$, *sacB*$^{+}$, Km^{r}	Laboratory collection
pRK2013	ColE1, RK2-Mob^{+}, RK2-Tra^{+}, Km^{r}	Laboratory collection
pK18*mobsacB*-Δ*cel9C*	Contains 500 bp upstream and downstream of *cel9C* to create an in-frame deletion; pMB1, *mob*$^{+}$, *sacB*$^{+}$, Km^{r}	This study
pK18*mobsacB*-Δ*cel5A*	Contains 500 bp upstream and downstream of *cel5A* to create an in-frame deletion; pMB1, *mob*$^{+}$, *sacB*$^{+}$, Km^{r}	This study
pK18*mobsacB*-Δ*cel5C*	Contains 500 bp upstream and downstream of *cel5C* to create an in-frame deletion; pMB1, *mob*$^{+}$, *sacB*$^{+}$, Km^{r}	This study
pK18*mobsacB*-Δ*cel5G*	Contains 500 bp upstream and downstream of *cel5G* to create an in-frame deletion; pMB1, *mob*$^{+}$, *sacB*$^{+}$, Km^{r}	This study
pCEL-	pMB1, Km^{r}	This study
pCEL1	pMB1, Km^{r}, *cel3B*$^{+}$	This study
pCEL3	pMB1, Km^{r}, *cel3B*$^{+}$, *cel5B*$^{+}$, *cel6A*$^{+}$	This study
pCEL6	pMB1, Km^{r}, *cel3B*$^{+}$, *cel5B*$^{+}$, *cel6A*$^{+}$, *lpmo10B*$^{+}$, *cbp2D*$^{+}$, *cbp2E*$^{+}$	This study

Tables S2 & S3 are provided in a separate Supplemental Data file (Excel)

Table S4. Complete list of all *C. japonicus* genes assessed in this study.

Gene Name	Locus ID	CAZy Family	Enzyme Activity (predicted)	E.C. # (predicted)	UniProt Accession
cel45A	CJA_0374	GH45	Endo-1,4-beta-glucanase	3.2.1.4	GUNB
cel5A	CJA_1462	GH5	Endo-1,4-beta-glucanase	3.2.1.4	GUNC
cel5B	CJA_2983	GH5	Endo-1,4-beta-glucanase	3.2.1.4	B3PCS3
cel5C	CJA_3369	GH5	Endo-1,4-beta-glucanase	3.2.1.4	B3PF55
cel5D	CJA_3010	GH5	Endo-1,4-beta-glucanase	3.2.1.4	B3PD52
cel5E	CJA_3337	GH5	Endo-1,4-beta-glucanase	3.2.1.4	B3PF23
cel5F	CJA_2959	GH5	Endo-1,4-beta-glucanase	3.2.1.4	B3PCP9
cel5G	CJA_2760	GH5	Endo-1,4-beta-glucanase	3.2.1.4	B3PBJ3
cel5H	CJA_0619	GH5	Endo-1,4-beta-glucanase	3.2.1.4	B3PJL3
cel6A	CJA_2473	GH6	Glucanase	3.2.1.-	B3PKK5
cel9A	CJA_2472	GH9	Endo-1,4-beta-glucanase	3.2.1.4	GUNA
cel9B	CJA_1633	GH9	Endo-1,4-beta-glucanase	3.2.1.4	B3PEQ9
cel9C	CJA_3804	GH9	Endo-1,4-beta-glucanase	3.2.1.4	B3PIS2
lpm10B	CJA_3139	AA10	LPMO	n/a	B3PDT6
cbp2D	CJA_2616	CBM2	n/a	n/a	B3PLJ6
cbp2E	CJA_2615	CBM2	n/a	n/a	B3PLJ5

Table S5. Current list of Cabrhydarte Binding Proteins (Cbps) in *C. japonicus*[a].

Locus ID	UniProt Accession	Gene Name	CBM Family	CBM(s) (# present)	Signal Peptide	Predicted Location
CJA_0007	B3PEM8	*cbp2A*	CBM2	2 (2)	non cleavable	outer membrane
CJA_3106	B3PDE8	*cbp2B*	CBM2	2	non cleavable	outer membrane
CJA_3107	B3PDE9	*cbp2C*	CBM2	2	cleavable	extracellular
CJA_2616	B3PLJ6	*cbp2D*	CBM2	2	cleavable	extracellular
CJA_2615	B3PLJ5	*cbp2E*	CBM2	2	cleavable	extracellular
CJA_2469	B3PKK1	*cbp2F*	CBM2	2	cleavable	extracellular
CJA_1191	B3PBX6	*cbp6A*	CBM6	6	non cleavable	outer membrane
CJA_0276	B3PH79	*cbp6B*	CBM6	6	cleavable	extracellular
CJA_3300	B3PEJ8	*cbp6C*	CBM6	6	none	cytoplasm
CJA_0831	B3PKS4	*cbp10*	CBM10	10	non cleavable	outer membrane
CJA_2869	B3PC55	*cbp26A*	CBM26	26	cleavable	extracellular
CJA_0778	B3PKM1	*cbp32A*	CBM32	32	none	cytoplasm
CJA_2657	B3PLN7	*cbp32B*	CBM32	32 (4)	cleavable	extracellular
CJA_2191	B3PJ79	*cbp33A*	CBM33[b] CBM5	5, 33	cleavable	extracellular
CJA_3139	B3PDT6	*cbp33B*	CBM33[b] CBM10	33, 10	cleavable	extracellular
CJA_0020	B3PEP0	*cbp35A*	CBM35	35	cleavable	extracellular
CJA_0559	B3PJ48	*cbp35B*	CBM35	35	cleavable	extracellular
CJA_0494	B3PIY3	*cbp35C*	CBM35	35	cleavable	extracellular

[a] Table adapted from DeBoy, *et al.* (2008) (PMID: 18556790).

[b] The CBM33 Family has been discontinued and now belongs to the Auxiliary Activity (AA) Classification for Lytic Polysaccharide Mono-Oxygenases (LPMOs). Current annotations for CJA_2191 and CJA_3139 are Lpmo10A and Lpmo10B, respectively.
